# Molecular mechanisms of human papillomavirus-induced tongue carcinogenesis: A systematic review

**DOI:** 10.1016/j.jobcr.2025.11.011

**Published:** 2025-12-10

**Authors:** Chamathsara Hewa Kodikarage, Menaka Batuwanthudawa, Kalpani Senevirathna, Wasala Mudiyanselage Kalpani Madhushika Ratnayake, Sivasuntharam Induijaa, Yovanthi Anurangi Jayasinghe, Kehinde Kazeem Kanmodi, Bogahawatte Samarakoon Mudiyanselage Samadarani Siriwardena, Ruwan Duminda Jayasinghe

**Affiliations:** a Department of Biochemistry, Faculty of Medicine, Uva Wellassa University, Badulla, 90000, Sri Lanka; b Department of Agricultural Biology, Faculty of Agriculture, University of Peradeniya, Peradeniya, 20400, Sri Lanka; c Department of Cosmetic Science, Faculty of Health Sciences, CINEC Campus, Malabe, 10115, Sri Lanka; d Department of Oral Medicine and Periodontology, Faculty of Dental Sciences, University of Peradeniya, Peradeniya, 20400, Sri Lanka; e Department of Oral Medicine and Periodontology, SIMATS Deemed University, Chennai, Tamil Nadu, India; f Department of Research, University of Puthisastra, Phnom Penh, Cambodia; g School of Health and Life Sciences, Teesside University, Middlesbrough, England, UK; h Department of Preventive and Community Dentistry, University of Rwanda, Kigali, Kigali, Rwanda; i Campaign for Health and Neck Cancer Education (CHANCE) Programme, Cephas Health Research Initiative Inc, Ibadan, Nigeria; j Department of Oral Pathology, Faculty of Dental Sciences, University of Peradeniya, Peradeniya, Sri Lanka; k RAK Collage of Dental Sciences, RAK Medical and Health Sciences University, UAE

**Keywords:** Human papillomavirus, Tongue cancer, Molecular mechanisms, Epigenetics, Tumour suppressors

## Abstract

**Background:**

Human papillomavirus (HPV) infection has emerged as a significant etiological factor in tongue cancer, particularly among individuals without conventional risk factors such as tobacco or alcohol use. Understanding the molecular mechanisms underlying HPV-induced tongue carcinogenesis is critical for advancing targeted interventions.

**Objective:**

To systematically review existing literature on genetic and epigenetic alterations in HPV-associated tongue cancer, with emphasis on viral oncoprotein interactions, disrupted cellular signalling pathways, and immune evasion mechanisms.

**Data sources:**

Electronic databases searched included PubMed, Scopus, Web of Science, Dentistry and Oral Sciences Source, and AMED.

**Methods:**

A systematic search using relevant MeSH terms was conducted to identify peer-reviewed studies involving human participants. Duplicates were removed, and studies were screened using the Rayyan software based on predefined inclusion and exclusion criteria. Quality assessment was performed using the Mixed Methods Appraisal Tool (MMAT).

**Results:**

The search yielded 3,140 articles, with 27 studies meeting the inclusion criteria after full-text screening and manual search. Quality assessment indicated that 96.3 % of studies (n = 26) had low risk of bias, while one study (3.7 %) had high risk. Key findings showed that HPV E6 and E7 oncoproteins interfere with tumor suppressor pathways (e.g., p53 and Rb), and activate PI3K/Akt, Wnt/β-catenin, and NF-κB signaling. Epigenetic alterations such as promoter hypermethylation, histone modification, and microRNA dysregulation were also implicated. HPV-positive tumours demonstrated immune evasion features.

**Conclusion:**

Despite progress in understanding HPV-related tongue carcinogenesis, further research is needed to explore tissue tropism and identify novel therapeutic targets.

**Prospero id:**

CRD42024593129.

## 1 Introduction

Oral cancer refers to a group of cancers affecting the lips, mouth (oral cavity), and throat (oropharynx).^1^ Oral cancer is one of the leading cancers and one of the leading causes of cancer-related deaths globally.^2^ Oral cavity cancer, a form of oral cancer, refers to malignancies arising in the hard palate, buccal mucosa, floor of the mouth, anterior two-thirds of the tongue, and retromolar trigones. In contrast, oropharyngeal cancer encompasses tumours of the posterior third of the tongue, tonsils, soft palate, and pharyngeal walls, whereas lip cancer refers to malignancy of the upper and/or lower lip(s).^3^, ^4^, ^5^ According to the Global Cancer Observatory (GLOBOCAN) estimates for 2022, approximately 389,485 new cases of lip and oral cavity cancers have been reported globally.^6^ This number is predicted to rise to 856,000 by 2035 owing to demographic changes.^7^ Most of the cancers occurring in the oral cavity are squamous cell carcinomas. Tongue is the most prevalent intraoral site for oral cancer worldwide, for instance, in the United States, 41.7 % of oral cavity squamous cell carcinomas occur on the tongue.^4^

The occurrence rates of tongue cancer differ substantially across different nations. For instance, the incidence rates of male tongue cancer in India stand at 6.5 per 100,000 people annually but France reports 8.0 cases per 100,000 people annually.^8^ The highest occurrence rates of oral cancer are observed in South-Central Asia and Oceania, with Papua New Guinea, Pakistan, and India leading in estimated incidence.^9^ This trend is particularly pronounced in India, which has earned the unfortunate moniker of “oral cancer capital of the world” due to its exceptionally high incidence of oral cancers. Among these, oral tongue squamous cell carcinoma (OTSCC) stands out as one of the two primary oral cavity subsites contributing to this alarming statistic.^10^

Oral cancer arises through the complex interaction of genetic, epigenetic, microbial, behavioral, and lifestyle factors, and is therefore considered a multifactorial disease.^10^ Established risk factors include tobacco use, areca nut chewing, alcohol consumption, chronic inflammation, ultraviolet radiation exposure (particularly in lip cancer), and *Candida* species, immunosuppression, hereditary susceptibility, and dietary habits.^9^ The etiology of tongue cancer differs between the anterior two-thirds and posterior one-third of the tongue.^9^ Traditionally, tobacco and alcohol have been regarded as the predominant causes; however, emerging evidence suggests a shift in etiological patterns. For instance, a study from India reported that 86.4 % of early-stage tongue squamous cell carcinoma patients had never consumed alcohol, and 49.3 % had no history of tobacco use, underscoring the possibility of alternative risk factors in certain populations.^10^ In the clinical setting, HPV testing focuses on 14 high-risk genotypes with carcinogenic potential, including HPV-16, 18, 31, 33, 35, 39, 45, 51, 52, 56, 58, 59, 66, and 68.^11^ Among these, HPV-16 is the predominant genotype, accounting for approximately 85–96 % of HPV-positive oropharyngeal cancer cases. HPV-18 also contributes significantly, particularly in cancers of the posterior tongue and oropharynx, although the role of high-risk HPV in anterior tongue carcinogenesis remains less clear.^10^^,^^11^ In contrast, low-risk HPV types (LR-HPV), most notably HPV-6 and HPV-11, are typically linked to benign conditions such as anogenital warts and recurrent respiratory papillomatosis, the latter being a rare but debilitating disease that reduces quality of life.^12^ Globally, HPV prevalence in tongue cancer varies widely, ranging from 0 % to 51.2 % depending on the region.^12^, ^13^, ^14^ According to GLOBOCAN 2022, oropharyngeal cancer is the 24th most common cancer worldwide, with an estimated 106,400 new cases annually, nearly 70 % of which are attributable to persistent infection with high-risk HPV strains.^13^

Integration of the HPV genome into the host chromosome is an important step in the HPV-induced carcinogenesis process.^15^ The carcinogenic potential of HPV 16 and 18 stems from their E6 and E7 proteins which block p53 and Rb tumour suppressors respectively.^16^ The host DNA develops mutations while cells continue uncontrolled growth.^17^ The E6 and E7 oncoproteins disrupt tumour-related signalling pathways including Wnt/β-catenin, PI3K/Akt and NF-kB which leads to the dysregulation of essential molecular pathways in host cells through interactions with critical cellular components that control cell cycle progression and result in malignant transformation.^18^^,^^19^ While HPV-driven oncogenesis involves common mechanisms across sites, such as E6 and E7 oncoproteins targeting p53 and Rb, site-specific factors in the oral tongue may alter these pathways. Understanding these mechanisms is vital for improving diagnosis, treatment, and prevention of HPV-associated tongue cancer. The existence of cancer stem cells (CSCs) within a tumour is a major contributor to therapy resistance, recurrence, and metastasis, leading to poor survival rates.^20^ New strategies, such as the use of valproic acid at clinically safe concentrations for tongue cancer cells, have been shown to be effective in reducing tumour cell viability as well as the migration potential of tongue cancer cells.^21^

There is currently a lack of consensus in the literature about the role of HPV infection as a predictive factor for tongue cancer. Not all tongue cancers may have the same prognosis as HPV positive oropharyngeal malignancies.^14^^,^^22^ In addition, the relationship between HPV infection and p16 upregulation, a biomarker used to identify HPV in oropharyngeal squamous Cell carcinoma (OPSCC), appears to be less accurate in tongue tumours.^14^

Novel approaches in genomic and epigenomic analysis have also enabled a fresh insight into the molecular differences in HPV associated tongue carcinogenesis.^23^ A few examples of these molecular differences are modifications in signalling networks, DNA repair and immune response.^23^ Since research on the immune system and tumour microenvironment in HPV-related tongue carcinogenesis is scarce, it is important to continue studying these areas as understanding the immunological signature of tongue malignancies that are HPV-positive may help identify new treatment targets. Overall, the molecular mechanisms of HPV-induced tongue carcinogenesis are complex and are not yet fully understood. There is a significant knowledge gap regarding the processes by which HPV causes tongue cancers and why the virus has a predilection for oropharyngeal tissues.^24^ This systematic review aims to synthesize current evidence on the molecular mechanisms of HPV-associated tongue cancer to guide future research and clinical practice. It seeks to identify knowledge gaps, improve early diagnosis and targeted therapies, enhance prevention strategies, and support patient education. By integrating multidisciplinary research, the review encourages collaboration and advances in the field.

## 2 Method

### 2.1 Protocol and registration

In order to identify recent empirical data on the molecular mechanisms underlying HPV-induced tongue carcinogenesis, the current systematic review was planned and carried out in accordance with the Cochrane Handbook for Systematic Reviews of Interventions^25^, ^26^ and the Preferred Reporting Items for Systematic Reviews and Meta-Analyses (PRISMA) guidelines.^27^ The protocol was registered with the International Prospective Register of Systematic Reviews (PROSPERO) (ID Ref: CRD42024593129).

### 2.2 Search strategy

Literature searches were conducted using five major electronic databases, namely PubMed, Scopus, Web of Science, Dentistry and Oral Sciences Source, and AMED (The Allied and Complementary Medicine Database) to identify studies published from 2014 to 2024. The most pertinent search queries were chosen using medical subject headings (MeSH) terms. To ensure a thorough exploration, specific keywords such as ‘Human papilloma’, ‘HPV’, ‘tongue’, ‘oral’, ‘cancer’, ‘carcino∗’, ‘malignant’, ‘molecular’, ‘pathogen’, ‘oncogene’, ‘gene’, ‘express’, ‘p53’, ‘pRb’, ‘pathway’ along with Boolean operators (“AND" and "OR"), and truncations (“∗") were employed. The search strings used are outlined using the PEO (P – population; E − exposure; O – outcome) framework in Table S1–S4 in the additional file [see Additional File 1]. Complete coverage was assured through a manual forward-backward search of the bibliographies of all the included literature.

### 2.3 Eligibility criteria

The inclusion and exclusion criteria of this systematic review are identified below.

#### 2.3.1 Inclusion criteria

• Peer-reviewed journal literature
• Studies focused on the molecular mechanisms involved in HPV-driven carcinogenesis of the both anterior and posterior tongue.
• Adopting an empirical research design (including case series with a minimum sample size of five patients).
• Investigating HPV-induced tongue carcinogenesis in humans.
• Published between 2014 and 2024 to capture most recent evidence.

#### 2.3.2 Exclusion criteria

• Literature published in non-peer‐reviewed journals, in books, book chapters and webpages.
• Secondary research literature, including review articles, editorials, letters, and commentaries.
• Literature not available in English.
• Literature whose full text is not accessible.
• Literature not specifically addressing HPV-induced tongue carcinogenesis.
• Literature focusing on non-molecular aspects of tongue cancer.

### 2.4 Screening and study selection

One reviewer (KKK) performed the literature search of the database. Using Rayyan software,^28^ screening was done in three steps: duplicate elimination, title and abstract screening, and full-text screening. After eliminating duplicates, the titles and abstracts of the remaining literature were examined by four independent reviewers (CHK, KS, MB, and WMKMR) to identify those meeting the predefined inclusion criteria. After filtering according to the titles and abstracts, the same reviewers then reviewed the full texts of the remaining articles that were potentially eligible. Discussion with a second reviewer (RDJ) was used to resolve any disagreements in article eligibility. The final list of articles was agreed upon by consensus among all reviewers.

### 2.5 Data extraction and synthesis

Six reviewers (CHK, MB, KS, WMKMR, SI, and YAJ) independently extracted data using a data extraction sheet. This sheet was initially tested, and then further refined and clarified through discussions with the research team before the actual extraction process began. The following information was extracted from each study: author details, publication year, study design, ratio of HPV-positive to HPV-negative cases, sample characteristics, tongue subsites, case distribution across these subsites, inclusion of other oral sites, if applicable, along with their specific locations and case numbers, investigated molecular mechanisms, genes or proteins of interest, implicated signalling pathways, experimental methodologies, control measures implemented, reported molecular alterations, and potential biomarkers identified. Additionally, study limitations, key findings, and conclusions were extracted to provide a holistic view of the scope and outcomes of each study. The data synthesis process was done using the narrative synthesis approach due to the heterogeneity in the design of the included literature.

### 2.6 Methodological quality assessment

Assessment of study quality was conducted independently by six reviewers (CHK, KS, MB, SI, WMKMR, YAJ). The evaluation process started with determining the study design. The Mixed Methods Appraisal tool (MMAT) served as the instrument for evaluating the quality of quantitative studies retrieved from the search.^29^ The MMAT assessment criteria consist of seven components which evaluate each study category. Studies received their percentage scores based on the number of criteria they fulfilled. Discrepancies were resolved through discussion.

### 2.7 Handling of potential cohort overlap

Several studies from Karolinska University Hospital appeared to involve overlapping patient populations, as suggested by similar recruitment dates, patient demographics, and author groups. Due to incomplete reporting, the exact degree of overlap could not be confirmed in all cases. As no quantitative pooling of patient numbers was conducted, these studies were treated as separate analyses of potentially overlapping datasets, with caution applied in interpreting the frequency of findings.

## 3 Results

### 3.1 Search outcome

**Fig. 1:**
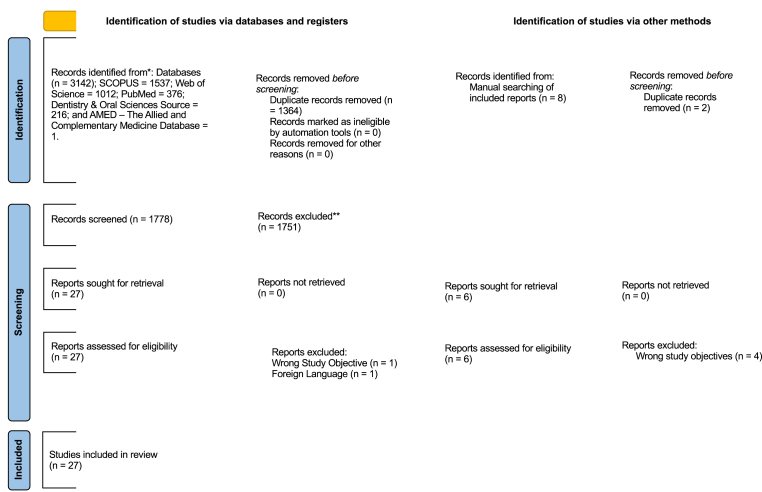
*SEQ Figure \∗ ARABIC 1:PRISMA flowchart diagram. PRISMA, Preferred Reporting Items for Systematic Reviews and Meta-analysis*.

A total of 3,142 studies were retrieved by searching across five databases (SCOPUS = 1,537, Web of Science = 1,012, PubMed = 376, Dentistry and Oral Science Source = 216, and AMED (The Allied and Complementary Medicine Database = 1). Duplicates (n = 1364) were removed to retain 1778 articles for title and abstract screening. After evaluating the titles and abstracts, a total of 27 articles were shortlisted for full-text screening, and 2 articles were excluded during full text screening as one was not published in English and the other was with a wrong study objective. Subsequently, two articles were included in the manual search of their bibliographies of the included articles. Therefore, 27 articles that met the inclusion criteria were retained for evaluation (Fig. 1).

### 3.2 Study designs

All included articles (n = 27) adopted a quantitative, nonrandomized study design. Among them, only 85.2 % (n = 23/27) adopted a cohort study design,^14^^,^^30^, ^31^, ^32^, ^33^, ^34^, ^35^, ^36^, ^37^, ^38^, ^39^, ^40^, ^41^, ^42^, ^43^, ^44^, ^45^, ^46^^,^^47^, ^48^, ^49^, ^50^, ^51^ whereas the remaining articles (n = 4/27; 14.8 %) adopted a case-control study design.^52^, ^53^, ^54^, ^55^ Of the included articles that adopted a cohort study design, the majority (n = 18/23; 78.3 %) were retrospective studies,^14^^,^^30^, ^31^, ^32^, ^33^, ^34^, ^35^, ^36^, ^37^, ^38^, ^39^, ^40^, ^41^, ^42^, ^43^, ^44^, ^45^, ^46^ and only 21.7 % (n = 5/23) were prospective studies.^47^, ^48^, ^49^, ^50^, ^51^

### 3.3 Risk of bias assessment outcomes

Out of the total 27 articles, 26 articles (96.3 %) were of above-average quality, while only one article (3.7 % of total articles) was of below average quality. Among the 26 articles that were scored above average, two articles were scored as 7/7,^32^^,^^41^ eleven as 6/7,^30^^,^^31^^,^^33^^,^^35^^,^^37^^,^^38^^,^^42^^,^^45^^,^^52^^,^^53^^,^^50^ eleven as 5/7,^14^^,^^36^^,^^39^^,^^40^^,^^43^^,^^44^^,^^46^^,^^47^, ^54^, ^55^^,^^51^ two as 4/7,^34^^,^^48^ and one as 3/7.^49^

### 3.4 Publication type and publication trend by year

All included articles were published between 2014 and 2024. Among these, two were published in 2021,^30^^,^^31^ two in 2020,^32^^,^^40^ five in 2018,^33^^,^^35^^,^^38^^,^^52^^,^^53^ three in 2017,^36^^,^^42^^,^^47^ two in 2016,^37^^,^^48^ seven in 2015,^34^^,^^39^^,^^44^^,^^46^^,^^55^^,^^49^^,^^50^ five in 2014,^14^^,^^43^^,^^45^^,^^54^^,^^51^ one in 2019.^41^

### 3.5 Sub-site of the tongue

The majority of included articles (55.56 %) focused on the posterior tongue, specifically the base of the tongue, to investigate the molecular mechanisms of HPV-induced tongue carcinogenesis.^30^, ^31^, ^32^, ^33^, ^34^, ^35^, ^36^, ^37^, ^38^^,^^41^^,^^43^, ^44^, ^45^, ^46^^,^^47^ The studies on the anterior tongue were less frequent but were included to assess the consistency of findings across the literature and to explore potential differences in molecular mechanisms when HPV is present in the anterior tongue. Of the articles examining the anterior tongue, 11.11 % considered lateral borders,^14^^,^^39^^,^^47^ 7.4 % included ventral sites, 7.4 % examined dorsal sites,^14^^,^^39^ and 3.7 % (one article) used the tip of the tongue.^14^ Notably, a significant portion (29.63 %) of articles did not specify the exact site of the tongue.^40^^,^^42^^,^^52^^,^^53^^,^^55^^,^^49^, ^50^, ^51^ This distribution highlights the predominant research interest in HPV-related tongue cancer at the base of the tongue, while also revealing the less frequent but varied studies on anterior tongue sites. The inclusion of anterior tongue studies allows for a comprehensive comparison of molecular mechanisms across different tongue regions in the context of HPV-induced carcinogenesis.

### 3.6 Sample size

A total of 4683 samples were analyzed across included studies, comprising 3,003 HPV-positive samples^14^^,^^30^, ^31^, ^32^, ^33^, ^34^, ^35^, ^36^, ^37^, ^38^^,^^40^, ^41^, ^42^, ^43^, ^44^, ^45^, ^46^^,^^53^^,^^47^, ^48^, ^49^, ^50^, ^51^, ^55^ and 1,680 HPV-negative samples.^14^^,^^31^, ^32^, ^33^^,^^36^, ^37^, ^38^, ^39^, ^40^, ^41^, ^42^, ^43^^,^^45^^,^^46^^,^^53^^,^^47^, ^48^, ^49^, ^50^, ^51^, ^55^

### 3.7 The molecular mechanisms reported

Molecular mechanisms identified can be broadly categorised into five distinct categories namely gene mutations and alterations, epigenetic mechanisms, immune system and tumour micro-environment related mechanisms, signalling pathways related mechanisms and biomarkers discovery and identification tools.

Among gene mutations and alterations, key mutations which are responsible for cancer progression such as mutations in CDC27, BCLAF1, and AQP7,^30^ common oncogenic mutations such as PIK3CA, TP53, and FGFR3 and TP53,^36^ Copy number variations (CNVs) and loss of heterozygosity (LOH) in CASP8, RASA1, and NOTCH genes^55^ and Microsatellite instability (MSI) and genetic instability^54^ were reported. Some gene mutations and key alterations were related to oncogene activation and tumour suppressor inactivation. Some articles included HPV-mediated inactivation of tumour suppressors^43^ whereas AP-1 pathway involvement in relation to the oncogenic activation and tumour suppressors were mentioned in some of the reports.^50^ Out of the epigenetic mechanisms reported, DNA methylation and gene silencing and MicroRNA Regulation was significant. Under the DNA methylation and gene silencing, Hypermethylation of tumour suppressor genes such as DAPK1, LRPPRC, and ZNF471 were reported.^47^ miR-155, miR-185, and miR-193b were found to be differentially expressed in HPV-positive tumours.^33^ Some articles include regulation of STING and immune evasion because of downregulating miR-27a/b.^49^

Among immune system and tumour microenvironment related molecular mechanisms, different immune evasion strategies were reported in some articles. For instance, downregulation of antigen presentation such as lower expression of HLA class I molecules and antigen-processing proteins such as TAP2, LMP2, and LMP7 was reported.^46^ Mechanisms related to infiltration of immunosuppressive Tregs which are responsible for creating immunosuppressive environments were also reported.^49^ Some included molecular mechanisms related to inflammation and toll-like receptors (TLRs) such as over-expression of TLR4 and TLR9 and high levels of interferon signalling (IFN-β).^40^

Some signalling pathways such as growth factor and receptor signalling and NF-κB and AP-1 Signalling were reported. Under the growth factor and receptor signalling pathways, FGFR3 mutations which are responsible for poor prognosis in HPV-positive tumours^35^ and VEGF-driven angiogenesis mechanisms were reported.^38^ Additionally, some articles included information about c-Rel overexpression which has the ability of promoting tumour growth and invasion.^53^ Mechanisms related to the activation of the AP-1 complex (c-Jun/Fra-2) which contributes to the tumour aggressiveness were reported in some articles.^50^

More importantly, Potential biomarkers and diagnostic tools were found. Several proteins, including S100A7, IL1RN, and CXCL8, were identified as potential biomarkers for HPV-positive tumours.^41^ Furthermore, p16 overexpression was often used as a surrogate for HPV positivity.^14^ (see Table 1)

Different molecular and biochemical tools and techniques have been used to investigate molecular mechanisms. Immunohistochemistry (IHC) was performed in eight studies.^30^^,^^31^^,^^35^^,^^38^^,^^40^^,^^42^^,^^53^^,^^48^ Seven studies used PCR and qPCR techniques.^37^^,^^38^^,^^42^^,^^53^, ^54^, ^55^^,^^48^ DNA Methylation Analysis by Bisulfite sequencing and methylation-specific PCR was carried out in two articles.^42^^,^^47^ RNA Analysis by RT-PCR was performed in three studies.^41^^,^^42^^,^^55^ Sequencing was performed in some articles^30^^,^^47^ (Table 2).

**Table 1:** Summary of the study characteristics.

No.	Author (Year)	Study Design	Sample Size	Sample Characteristics				Study limitations	Sample Origin
				HPV cases Vs non-HPV cases	Sub site of the tongue and number of cases	Area of the tongue examined	Other characteristics		
01	Ährlund-Richter et al., 2021^30^	Cohort study (Retrospective)	45	HPV positive - 35	Base of tongue - 5	Posterior (The study focuses on squamous cell carcinomas in the base of tongue (BOTSCC) and tonsils (TSCC)).	No controls were used HPV-positive tonsillar and base of tongue squamous cell carcinoma (TSCC/BOTSCC)	Small number of patients, primarily because relapse is rare in HPV-positive OPSCC.	Sweden [Karolinska University Hospital, between 2000 and 2014.]
02	Zupancic et al., 2021^31^	Cohort Study (Retrospective)	72	HPV positive - 56 HPV negative - 16	Base of tongue - 72	Not specified.	There were no controls. BOTSCC and TSCC with and without HPV were both utilized.	Psoriasis expression had no prognostic effect in HPV DNA-negative BOTSCC patients. However, caution is needed due to the small sample size (16 patients). This pilot study did not include TSCC or other OPSCC patients.	Sweden [County of Stockholm, between 2000 and 2007; The patients were treated at Karolinska University Hospital, and the study was conducted at Karolinska Institutet in Stockholm, Sweden.]
03	Marklund et al., 2020^32^	Cohort Study (Retrospective)	932	HPV positive - 661 HPV negative −264 Undetermined - 7	Base of tongue - 261	Not specified (The study focuses on oropharyngeal squamous cell carcinomas (OPSCC), which includes cancers of the tonsils, base of tongue, and other oropharyngeal sites. While base of tongue carcinomas (BOTSCC) is mentioned, there is no specific discussion of anterior, posterior, or combined tongue areas).	There were no controls. BOTSCC and TSCC with and without HPV were both utilized.	Single-centre study with retrospectively collected clinical data. Overall survival was not affected by treatment technique. The TNM grades were assigned without regard to treatment modalities, using data gathered retrospectively. Potential influence of local treatment traditions on outcomes cannot be excluded.	Sweden [Stockholm, between 2000 and 2016.]
04	Bersani et al., 2018a^33^	Cohort Study (Retrospective)	168	HPV positive - 110 HPV negative - 52	Base of tongue - 162	Not specified (The study examined tonsillar and base of tongue squamous cell carcinoma (TSCC/BOTSCC). Specifically, it focused on cancers of the tonsils and base of tongue.)	There were no controls. BOTSCC and TSCC with and without HPV were both utilized.	The study was retrospective, with tumour samples from patients treated between 2000 and 2013. Treatment intensity increased, especially after 2008. High survival rates in HPV + TSCC/BOTSCC patients led to too few events to link miR expression with survival and treatment.	Sweden [ Karolinska University Hospital, between 2000 and 2013]
05	Ramqvist et al., 2018^38^	Cohort Study (Retrospective)	59	HPV positive - 42 HPV negative - 17	Base of tongue - 59	Not specified. (The study does not specify examining anterior or posterior regions of the tongue separately, but rather focuses on base of tongue cancer as a whole.)	There were no controls. BOTSCC and TSCC with and without HPV were both utilized.	The study included only 59 tumours, which increased the risk of random correlations, especially when analyzing HPV-positive and HPV-negative tumours separately. The cohort had heterogeneous treatment approaches and T-stage variations.	Sweden [Karolinska University Hospital in Stockholm; The patients were treated at this hospital between 2002 and 2011. The validation set included patients treated at the same hospital from 2000 to 2010.]
06	Bersani et al., 2018b^35^	Cohort Study (Retrospective)	115	HPV positive - 115	Base of tongue - Not differentiate the number to subside altogether 115 HPV + TSCC/BOTSCC	Not specified. (It focused on the base of tongue (ICD-10 code C01.9) and tonsillar regions (ICD-10 codes C09.0, C09.1, C09.8, C09.9)	No controls were used. Human participants with HPV + TSCC/BOTSCC were used.	Only three common FGFR3 mutations were tested, so rare mutations, amplifications, or translocations may have been missed. The small number of HPV + TSCC/BOTSCC cases may limit conclusions about FGFR3 mutations and survival. The follow up duration may be relatively short to make conclusions about the long term prognostic significance of FGFR3 mutations.	Sweden and Romania [Karolinska University Hospital in Stockholm, 2000–2011; Some HPV-negative samples were obtained from Iasi, Romania]
07	Khowal et al., 2018^52^	Case control study	16	Not specified	Not specified OSCC-Well Differentiated (OSCC-WD) localized in tongue - 3 Dysplasia in tongue - 1 case	Not specified. (The study only states that the case involved a single ulceration on lateral (left) margin of tongue which later increased to five ulcers.)	Human participants with Oral Squamous Cell Carcinoma (OSCC) and other oral pathologies were used Even though the sample size is 16, each provides one diseased oral lesion (POB) and one adjacent normal tissue (ANT) sample. (16 POB samples + 16 ANT samples) OSCC-Well Differentiated (OSCC-WD) lesions were localized on the tongue for some patients. In addition to that OSCC-Moderately Differentiated (OSCC-MD) lesions were found in other oral regions such as the tonsillar fossa, gingivo-buccal sulcus, and alveolar ridge of the mandible, but no specific mention of lesions on the tongue for these cases.	The study included a small group of OSCC patients without addictive habits, limiting its generalizability. Results were validated in a small cohort, so larger studies are needed. Only proteins and transcripts were analyzed, missing factors like epigenetics and non-coding RNAs. There was no follow-up data to evaluate the long-term prognostic value of the identified biomarkers.	India [Jamia Hamdard University in New Delhi]
08	Gupta et at. 2018^53^	Case control study	100	HPV positive - 14 HPV negative - 36	Not specified. It primarily refers to the overall tongue squamous cell carcinoma (TSCC) Precancerous lesions - 20 Cancer - 50 Normal adjacent controls - 30	The study examined both the base of tongue and mobile tongue/other sites of tongue. Specifically, out of 50 tongue cancer cases: 22 (44 %) were from the base of tongue - 28 (56 %) were from the mobile tongue and other sites of tongue. The study also provides a breakdown of these sites in relation to HPV status: Among HPV-positive cases, 85.7 % were from the base of tongue and 14.3 % from mobile tongue/other sites Among HPV-negative cases, 27.8 % were from the base of tongue and 72.2 % from mobile tongue/other sites.	Normal adjacent tissue samples were used as controls for comparison with cancerous and precancerous tissues. Human participants with the study examined tongue squamous cell carcinoma (TSCC) cases with tissue samples from 20 precancerous lesions, 50 cancer cases, and 30 adjacent normal controls, including 14 HPV16-positive and 36 HPV-negative cases.	The study included only 100 tissue samples, which may not be enough for broad conclusions. It was conducted at a single hospital, limiting generalizability. The focus was on c-Rel and NF-κB pathways, leaving other pathways unexplored. There was no additional functional validation in clinical settings or animal models.	India [Department of ENT Surgery of Dr. Ram Manohar Lohia Hospital, New Delhi]
09	Minami et al., 2017^42^	Cohort Study (Retrospective)	127	HPV positive - 7 HPV negative - 120	Not specified	The study specifically examined mobile tongue cancer (MTC) which typically refers to the anterior two-thirds of the tongue.	Human participants with Mobile Tongue Cancer (MTC) were used. Healthy control population for comparison of HPV DNA presence were used Non-HPV-positive tumour cases for comparison with HPV-positive tumours were used.	The retrospective design may introduce bias. FFPE samples were used, with no frozen tumour specimens for better analysis. A small number of HPV positive cases constrained the statistical power for survival analysis. Multivariable analysis was not possible due to the low number of cases.	Japan [Saitama Medical School International Medical Centre, between 2007 and 2015]
10	Bhat et al., 2017^47^	Cohort Study (Prospective)	20	HPV positive - 11 HPV negative - 9	Base of tongue - 7 (4 HPV-positive and 3 HPV - negative) Anterior/lateral regions - 10 (5 HPV-positive and 5 HPV-positive), Not specified for 3 cases	Not specified. (The study mentions that some samples were from the tongue base (57 % HPV positive) and others from anterior or lateral regions (50 % HPV positive), but does not provide a breakdown of how many samples came from each area.)	Human participants with squamous cell carcinoma of the tongue (SCCT) Matched with normal tissues as controls	To further confirm the diagnostic and prognostic importance of the discovered methylation signatures, larger case cohorts are needed. Although genes like LRPPRC, RAB6C, and ZNF471 were analyzed, further studies are needed to understand their role in SCCT.	India [Kasturba Medical College, Manipal, Karnataka]
11	Bersani et al., 2017^36^	Cohort Study (Retrospective)	325	HPV positive - 279 HPV negative - 46	Base of tongue - 87	Not specified.	Human participants with tonsillar and base of tongue squamous cell carcinoma were taken. HPV negative TSCC/BOTSCC cases were used a s controls.	This study used a retrospective methodology and only evaluated hotspot mutations in 50 oncogenes and tumor suppressor genes. The number of HPV + HNCUP cases was limited to 19.	Sweden [Karolinska University Hospital in Stockholm, Sweden between 2000 and 2011.]
12	Zafereo et al., 2016^48^	Cohort Study (Retrospective)	460	HPV positive - 10 HPV negative - 156	Not specified - 147 (65 HPV PCR-positive, 82 HPV PCR-negative)	Not specified.	Human participants with squamous cell carcinoma of the oral cavity (SCCOC) were used p16/HPV testing has been performed for all 460 samples to check HPV status. HPV-negative SCCOC cases were used as control group	Not all patients were tested for HPV using all three assays. Only high-risk HPV types 16 and 18 were tested using PCR. Because of DNA fragmentation in paraffin-embedded specimens, HPV related SCCOC may be underreported. There was no HPV E6/7 mRNA expression assay to confirm as a gold standard.	United States [At a single tertiary cancer center; patients with squamous cell carcinoma of the oral cavity (SCCOC) were recruited at the University of Texas MD Anderson Cancer Center. ]
13	Garnaes et al., 2016^37^	Cohort study (Retrospective)	797	HPV positive - 452 HPV negative - 345	Base of tongue - 203 cases	Not specified.	Human participants with palatine tonsillar and base of tongue squamous cell carcinomas HPV negative tumours were used as controls Among all HPV positive samples, BSCC, STSCC and NSTCC represent 106, 267 and 79 respectively.	Clinical information was missing for 38 patients, including smoking status, TNM stage, treatment information, and registration in the Danish Civil Registration System. These patients were excluded from the study due to inability to collect the missing data. The study notes technical difficulties in performing E6/E7 RNA detection on archival tissue for large-scale studies. The study is based on a Danish population with a high HPV prevalence, which may limit its applicability to other populations with different HPV rates or demographics.	Eastern Denmark [Between 2000 and 2010]
14	Ramqvist et al., 2015^34^	Cohort Study (Retrospective)	117	HPV positive - 117	Base of tongue - 19	Not specified. (The study does not specify examining anterior or posterior regions of the tongue separately, but rather focuses on base of tongue cancer as a whole.)	Human participants with HPV16 DNA and E7 mRNA positive tonsillar and base of tongue squamous cell carcinoma (TSCC and BOTSCC) HPV negative cases were not specified. Cell lines namely SiHa, UM-SCC-47, UPCI-SCC-154, UM-SCC-14 and water samples were used as negative controls	The study had a retrospective design. FFPE samples were used, which may affect mRNA analysis. The sample size was relatively small. mRNA analysis was suboptimal due to the use of FFPE samples.	Sweden [Karolinska University Hospital in Stockholm, between 2000 and 2011]
15	Liang et al., 2015^49^	Cohort Study (Prospective)	100(111 with 11 cell lines)	HPV positive - 25 HPV negative - 25	Not specified. All were TSCC	Not specified.	HPV-positive and HPV-negative tongue squamouse cell carcinoma samples from human subjects. The cell lines used include HSC-3, Scc-4, Scc-1, HB, Scc-14a, Scc-14b, OSCC-3, and Cal-27. In addition, baseline activity was determined using untreated cell lines, PBMCs from healthy donors without STING activators, HPV-TSCC samples, and about 100 ml of fresh peripheral blood from healthy donors.	There is lack of longitudinal data, so tracking the progression of TSCC over time is not possible. There is a lack of robust in vivo models for HPV + TSCC. The sample size is relatively small. The study does not directly link its findings to patient outcomes, such as survival or therapy response. There is variability in patient demographics.	China [Nanjing Stomatology Hospital between 2003 and 2011. ]
16	Sgaramella et al., 2015^39^	Cohort Study (retrospective)	174	Although 36 cases were p16-positive, no HPV DNA was detected. All 109 cases are considered non-HPV	Lateral border of mobile tongue - 67 % Ventral side of the tongue - 20 % Dorsal side of the tongue - 2 % Widespread/location cannot determine properly - 11 %	Not specified. (The study states that the majority of tumours (67 %) were localized on the lateral border of the mobile tongue, 20 % on the ventral side, and 2 % on the dorsal side. In 11 % of cases, lesions were so widespread that it was not possible to state the prime localization on the mobile tongue. The study focused on squamous cell carcinoma of the mobile tongue in general.)	109 samples of primary tongue squamous cell carcinoma. samples were formalin-fixed, paraffin-embedded biopsies. Cervical epithelium sample with known HPV16 infection was used as a positive control	The study found no HPV16 DNA in the TSCC samples and did not focus on other HPV subtypes. In situ hybridization is not effective for detecting the virus when the load is very low. The study did not fully explore the molecular mechanisms behind p16 overexpression in TSCC. The follow-up period was inconsistent.	Sweden and Italy [Clinical Pathology, Umeå University Hospital, Sweden and Second University of Naples, Multidisciplinary Department of Medical, Surgical and Dental Specialties, Naples, Italy]
17	Tertipis et al., 2015a^44^	Cohort Study (Retrospective)	315	All are HPV positive (315)	Base of tongue - 112	Not specified.	Tumour Biopsies from Tonsillar Squamous Cell Carcinoma (TSCC) and Base of Tongue Squamous Cell Carcinoma (BOTSCC) were used All HPV positive 315 consist of 197 training cohort and 118 validation cohort.	This limits the ability to establish causal relationships between biomarkers and clinical outcomes. It does not include HPV-negative tumours for direct comparison. The research was carried out at one institution.	Sweden [Karolinska Institutet and Karolinska University Hospital in Stockholm, between 2000 and 2011]
18	Gupta et al., 2015^50^	Cohort Study (Prospective)	100	HPV positive - 14 HPV negative - 36 (all include tongue cancer)	Not specified (Tongue)- 50	The study examins different areas of the tongue, including: 1. Base of tongue: 85.7 % of HPV-positive cases were located in the base of the tongue. 2. Mobile tongue: Some cases were located in the mobile tongue, though a specific percentage is not provided. 3. Other sites of tongue: The study does not specify which areas. The study does not provide a clear breakdown of anterior vs posterior tongue regions. It primarily distinguishes between base of tongue and mobile tongue/other sites.	Cancer patients, having tonsillar and base of tongue squamous cell carcinoma, were involved in the study. The sample comprised of pre-cancerous (n = 20), cancerous (n = 50) and adjacent normal controls (n = 30). Adjacent normal cells were used as positive controls	Relatively low sample size	India [Department of ENT Surgery of Dr. Ram Manohar Lohia Hospital in New Delhi]
19	Panda et al., 2015^55^	Case- Control Study	50	HPV positive - 23 HPV negative - 16	Not specified (Tongue) - 50	Not specified.	Oral tongue squamous cell carcinomas (OTSCC) were used Fifty treatment-native patients diagnosed with oral tongue squamous cell carcinoma (SCC). Blood and/or adjacent normal tissue samples from the same patients to serve as controls for tumour specimen analysis.	Relatively low sample size	India [Mazumdar Shaw Medical Centre]
20	TSIMPLAKI et al., 2014^51^	Cohort Study (Prospective)	53	HPV positive - 6 HPV negative - 47	Not specified (Tongue)- 53	Not specified.	Oral tongue squamous cell carcinoma (SCC) was used No separate control group was used	Relatively low sample size	Greece [St. Savvas Regional Anticancer Oncology Hospital of Athens, between May 2012 and May 2013]
21	Adduri et al., 2014^54^	Case-control study	121	Can't retrieve the specific information as the addition data file is unable to find	Can't retrieve the specific information as the addition data file is unable to find	Not specified.	Squamous cell carcinoma of tongue was used There were 121 tumor/normal sample pairings in all (all oral tongue; 106 were recently removed, and 15 were preserved). No separate control group was used	Relatively low sample size	India [Three hospitals in Hyderabad]
22	Ramshankar et al., 2014^14^	Cohort Study (Retrospective)	167	HPV positive - 75 HPV negative - 81	Lateral Border - 138 Tip - 3 Dorsum - 5 Ventral Aspect - 10	Not specified.	Oral tongue squamous cell carcinoma was used No separate control group was used .	The findings are based on a single cohort, and no validation in independent or larger cohorts has been conducted to confirm the generalizability of the results.	India [from 1995 to 2007 at a tertiary cancer facility in Chennai, South India]
23	Tertipis et al., 2014a^43^	Cohort study (Retrospective)	278	HPV positive - 207 HPV negative - 71	Base of tongue - 83	Not specified.	Formalin-fixed, paraffin-embedded tumour biopsies of patients were taken Normal lymphoid tissues were used as the control group	The retrospective nature of the study. There were fewer HPV-negative cases in the study. It directly represents OSCC patients in Sweden.	Sweden [Karolinska University Hospital in Stockholm, between 2000 and 2007]
24	Tertipis et al., 2014b^45^	Cohort study (Retrospective)	425	HPV positive - 305 HPV negative - 120	Base of tongue - 118	Not specified.	The research used paraffin embedded tissue samples that were formalin fixed (FFPE). Water served as the negative control. A fresh blood sample that was positive for the HLA-A∗02 gene served as the positive control.	The retrospective nature of the study. There were fewer HPV-negative cases and females. The patients in this study were mostly from one age group, generally older.	Sweden [Karolinska University Hospital in Stockholm, between 2000 and 2009]
25	Haeggblom et al., 2019^41^	Cohort study (Retrospective)	24	HPV positive - 13 HPV negative - 11	Base of tongue - information on the # of samples has not been stated	Not specified.	Haematoxylin/eosin stained FFPE tumour sections were used Information was not stated about controls	High-grade dysplasia or carcinoma in situ tumor areas were frequently modest and, in certain situations, diminished with serial sectioning. Because FFPE tissue was used, the isolated RNA was in extremely small amounts and had been deteriorated. Because it performs well with these kinds of samples, a Nano String RNA panel was employed. RNA sequencing (RNAseq), on the other hand, would have offered more thorough information and improved understanding of the change from dysplasia to aggressive malignancy. Few patient samples were included because dysplasia is rare in HPV-positive tonsillar and base of tongue cancers.	Sweden [Karolinska University Hospital between 2007 and 2015]
26	Tertipis et al., 2015b^46^	Cohort study (Retrospective)	151	HPV positive - 101 HPV negative - 50	Base of tongue - 73	Not specified. (The study focuses on oropharyngeal cancers, particularly those originating in the base of tongue and tonsils.)	Formalin-fixed paraffin-embedded tumour biopsy slides were used Secondary Antibody was used as the negative control TAP2, LMP2, LMP7 gene-positive sample were used as the positive controls	Immunohistochemistry was used to measure TAP2, LMP2, and LMP7 expression, but methods like RNA sequencing or proteomics might have provided deeper insights. The study links APM component expression to clinical outcomes but does not fully explain the underlying mechanisms or causal relationships.	Sweden [Karolinska University Hospital in Stockholm, Sweden. 78 TSCC samples from patients diagnosed between 2000 and 2006 and 73 BOTSCC samples from patients diagnosed between 2000 and 2007 were included in the patient cohorts.]
27	Daskalopoulos et al., 2020^40^	Cohort Study (Retrospective)	63	HPV positive - 10 HPV negative - 53	Not specified	Not specified.	The study included oral tongue biopsy samples that had been diagnosed with either carcinoma in situ or invasive SCC by histopathology. No control was used in the study	The study has small sample sizes for histopathological subgroups and is retrospective. Technical biases in immunohistochemistry and the lack of alternative validation or strong clinical correlations with outcomes like survival or treatment response weaken the findings. The study also misses opportunities to explore the therapeutic implications of targeting TLRs, NF-kB, or IFN-β.	Greece [Department of Oral Medicine and Pathology, University of Athens]

**Table 2:** Summary of extracted findings.

Molecular mechanism investigated	Genes/Proteins interested (Potential Biomarkers)	Experimental Method Used	Key Findings	Reference
Comparison of genetic profiles of cases treated with curative intent, focusing on patients with and without relapse	CDC27, BCLAF1 and AQP7	Sequencing was performed on NovaSeq 6000 (Illumina, San Diego, CA, USA)	CDC27 deletions have only been found in the tumours 5/17 recurrence. CDC27 has been absent in non-recurrence cases Frequent mutations have occurred in CDC27, BCLAF1, and AQP7 among 26 mutated genes in ≥30 % of primary tumours, regardless of prognosis. CDC27 deletion (∼30 % of relapse/metastasis cases) has a prognostic potential Therefore, CDC17 can serve as a predictive marker for prognosis. There have been BCLAF1 and OVCH2 deletions as well as an OR2T35 substitution. Additionally, 26 genes were found to be mutated in over 30 % of cases.	^30^
Analyze the prognosis of psoriasin in BOTSCC, with an emphasis on HPV + BOTSCC.	Psoriasin	Immunohistochemistry	As for the patients with low levels of psoriasin expression the results were much better. p = 0.001 for overall survival (OS) p = 0.007 for disease-free survival (DFS) For HPV positive base of tongue squamous cell carcinoma patients (HPV + BOTSCC): OS: p < 0.001 DFS: p = 0.02 Psoriasin was a significant prognostic factor in both univariate and multivariable studies.	^31^
Relationships between overall survival (OS) and p16+ and tumor stage by OPSCC subsite.	p16+	Immunohistochemistry using monoclonal antibody (mAb) clone JC8	Patients who had p16+ other OPSCC had a worse overall survival (OS) than patients with p16+ TSCC/BOTSCC (p = 0.005). Comparable survival to patients with BOTSCC, TSCC, or p16-other OPSCC Patients with p16+ other OPSCC and TNM-8 stages I–II had a worse OS than those with p16+ TSCC/BOTSCC at the same stage (p = 0.02). Other OPSCC patients with low TNM-7 stage had a better OS than those with high stage (p = 0.019). There was no evidence of hazard discrimination in the TSCC/BOTSCC TNM-7 stage. The prognosis was better for OPSCC patients with HPV DNA and p16 positive OPSCC than for those with HPV DNA negative and p16 positive OPSCC.	^32^
MicroRNAs (miRs) 155, 185, and 193b were analyzed as possible prognostic indicators in TSCC/BOTSCC.	microRNAs (miRs) 155, 185 and 193b	Real-Time PCR	Tumor versus healthy tonsils: Reduced expression of miR-155. increased expression of miR-193b. Additionally, it is linked to low T-stage, high CD8^+^ TIL levels, HPV positive, and improved overall survival. HPV-negative patients had lower miR-185 levels and a worse survival tendency than those with HPV-positive patients. Among the 9 miRNAs, miRNA-185 was the only miRNA that was significantly related to survival (Cox regression). The present study suggests that miR-193b is related to more advanced T-stage, male gender, and reduced CD8^+^ TIL densities. It has no link with the patient's prognosis In HPV + patients, the combination of miR-155 and miR-185 predicted the outcome with an AUC of 71 %.	^33^
Immunoregulatory proteins and chemokines	VEGF	Olink multiplex immunoassays	HPV negative TSCC/BOTSCC shows different protein expression compared to HPV positive TSCC/BOTSCC with 34 proteins mostly immunoregulatory proteins and chemokines. The immune-related protein variations between HPV-positive and negative TSCC/BOTSCC indicate that HPV-positive cases have higher immune defense activity. Potential link between proteins and clinical outcomes Proteins were mainly angiogenesis and hypoxia related for HPV positive tumours. The correlation of VEGFA with clinical outcomes was also confirmed by immunohistochemistry. Angiogenesis related proteins may be potential therapeutic targets in HPV positive TSCC/BOTSCC.	^38^
FGFR3 expression with IHC. FGFR3 Mutation Detection	FGFR3 gene (mutations: p.R248C, p.S249C, p.K650Q), HPV16 (DNA, E7 mRNA), p16 (as a marker of HPV positivity)	Immunohistochemistry (IHC), PCR-Based Assays (Ion AmpliSeq Cancer Hotspot Panel v2, CAST-PCR), HPV Analysis (HPV DNA Status using PCR-based bead-based multiplex assay, p16 Overexpression using monoclonal antibodies through standard IHC.	CAST-PCR detected FGFR3 mutations in 10.1 % of HPV + TSCC/BOTSCC samples, with COSM715-p. S249C being the most common. The analysis showed that patients with higher FGFR3 expression achieved better 3-year disease-free survival (DFS) among HPV + TSCC/BOTSCC patients who had wild-type FGFR3 (p = 0.043). The analysis of the complete patient population revealed no significant variations in survival rates or overall survival between patients with FGFR3 mutations. FGFR3 expression and mutation status proved to be important prognostic indicators because patients with FGFR3 mutations in HPV + TSCC/BOTSCC experienced worse DFS.	^35^
HPV16 DNA presence and integration Proteomic profiling of serum and cellular samples Protein expression differences between diseased lesion (POB) and adjacent normal tissue (ANT). Identification of potential biomarkers through proteomic analysis.	Genes: HPV16 DNA (L1, E2, E6) Proteins: Serum and tissue proteins	HPV16 Assessment - PCR and nested PCR with HPV specific primers for HPV DNA detection, PCR for HPV integration status. Proteomic Analysis -One-dimensional SDS-PAGE for protein separation, Two-dimensional gel electrophoresis. MALDI-TOF MS for protein identification. Serum proteome and cellular proteome were analyzed.	OSCC diagnosis relies on visual and histopathological examination. Therefore, there is a need for better molecular tools for early diagnosis Potential therapeutic, diagnostic, and prognostic benefits were found for eight proteins implicated in oral carcinogenesis. When compared to nearby normal tissues, OSCC biopsies revealed notable proteomic variations, including modifications in serum proteome profiles. Certain protein changes, including those in the SCCA1, GIPC2-II, MRP L17, and IgM variable area in biopsies, were discovered in OSCC biopsies and serum. Serum levels of KRT1, SSFA2-IV, UPF0415, and DNAJC13. HPV16 Integration: HPV16 DNA integration in oral biopsies, suggesting its role in carcinogenesis. Upregulation of genes (DNAJC13, SSFA2-IV, UPF0415, GIPC2-II, MRPL17, IG-Vreg) and downregulation of KRT1 and SCCA1 in oral cancer and precancerous lesions. Proteins exhibited molecular weight differences, indicating post-translational modifications like glycosylation.	^52^
c-Rel transcriptional regulation, NF-κB signaling pathway, Cell migration, Cell invasion, Protein-protein interactions (PPIs)	c-Rel, NF-κB family proteins (p50, p65, p52, c-Rel, RelB), Fra-2	Electrophoretic Mobility Shift Assay (EMSA), Immunoblotting (Western Blot), siRNA Interference Assay, Matrigel Invasion Assay, Migration Assay (Cell Scratch Assay), String Analysis (Protein-Protein Interaction Prediction)	Both precancerous and malignant tongue tissues have overexpressed c-Rel, indicating that it contributes to the development of cancer. TSCC cells' capacity to invade and migrate was reduced when c-Rel levels were lowered, demonstrating that c-Rel aids in the spread of cancer cells. c-Rel influences the NF-κB pathway, which contributes to inflammation and cancer progression by promoting cell survival and proliferation. c-Rel expression was higher in HPV-negative TSCC cases, indicating it may behave differently in HPV-positive vs. HPV-negative cancers and it suggests HPV status may affect c-Rel's role in cancer progression. c-Rel upregulation regulates key genes that contribute to cancer cell proliferation, growth, spread survival, and invasion in TSCC cells, making it a potential target for treatment.	^53^
HPV DNA presence, p16 overexpression, p53 overexpression, HPV E6/E7 mRNA expression	p16 (CDKN2A) protein, p53 protein, HPV E6 and E7 genes	Immunohistochemistry (IHC) for detecting p16 and p53 expression., HPV DNA detection using real-time PCR to identify the presence of HPV., E6/E7 mRNA expression analysis to confirm HPV involvement.	Only 5.5 % of MTC cases were HPV-positive based on E6/E7 mRNA expression with a correlation to HPV types 16, 18, and 33. Therefore, HPV prevalence was low. 14.2 % of tumours over-expressed p16, but its correlation with HPV was weak. p16-positive tumours had significantly better cause-specific survival (CSS), regardless of HPV status. No notable difference in overall survival (OS) or CSS based on HPV DNA or E6/E7 status. p16 expression appears to be a prognostic marker independent of HPV status in MTC patients. p53 overexpression was found in 35.4 % of the tumours, 7.1 % had HPV DNA. p16 and CDKN2A Alterations: p16 overexpression was suggested to result from molecular mechanisms other than HPV, such as mutations, deletions, or methylation of the CDKN2A gene	^42^
DNA methylation	DAPK1, LRPPRC, RAB6C, ZNF471	Differential methylation hybridization (DMH) microarray, Bisulfite genomic sequencing (BGS), HPV typing by nested PCR and direct sequencing, Immunohistochemical staining	DAPK1, LRPPRC, RAB6C, and ZNF471 promoter hypermethylation in SCCT DNA methylation and gene expression have an inverse relationship. HPV prevalence of 55 % in SCCT cases Reduced DAPK1 expression in SCCT tissues	^47^
Mutations in cancer-related genes	PIK3CA, TP53, FGFR3, FBXW7, PTEN, CDKN2A	Next-generation sequencing using Ion AmpliSeq Cancer Hotspot Panel v2	HPV-positive tumours have fewer mutations per tumor than HPV-negative tumours. PIK3CA most common mutation in people with HPV BOTSCC/TSCC The most common mutation in HPV-negative BOTSCC/TSCC In HPV-positive TSCC/BOTSCC, FGFR3 mutations, particularly the S249C variant, were associated with a poorer prognosis. In addition, mutations were found in FBXW7, PTEN, CDKN2A, IDH2, ABL1, BRAF, EGFR, NOTCH1, and PTPN11.	^36^
HPV involvement in SCCOC	p16, HPV 16/18 E6/E7	p16 immunohistochemistry (IHC), PCR-based HPV 16/18 E6/7 DNA testing, HPV in situ hybridization (ISH)	Very low incidence of genuine HPV driven SCCOC (6 % or less) Over expression of p16 in 30 % of tumours, which is not necessarily related to HPV status. Hence, there is a very poor correlation between p16 IHC and HPV PCR/ISH results. Similar clinical features and treatment responses between HPV positive and negative cases.	^48^
HPV-related carcinogenesis	p16, pRB, p53	HPV DNA PCR, p16 immunohistochemistry	Prognosis of BSCC and STSCC patients was better with dual positivity for HPV DNA and p16. HPV+/p16+ tumours had worse overall survival than HPV-/p16- tumours. HPV DNA/p16 testing was a better prognostic marker than either test alone for BSCCs and STSCCs. p16: Overexpression in HPV positive tumours.	^37^
HPV16 E2, E5, and E7 mRNA expression	HPV16 E2, E5, E7, HLA class I	Multiplex bead-based assay for HPV16 E2, E5, and E7 mRNA detection, PCR for HPV DNA detection, Immunohistochemistry for p16, HLA class I, and CD8^+^ TIL analysis	Almost all HPV16 DNA positive tumours expressed E7 mRNA (92 %). 64 % of E7 mRNA positive tumours also expressed E2 and E5 mRNA Variability in E2 and E5 mRNA expression among HPV16 positive tumours. The absence of E2 mRNA was associated with worse 3-year relapse-free and progression-free survival. E5 mRNA expression did not correlate with clinical outcome. No correlation of E2 or E5 mRNA expression with HLA class I expression and CD8^+^ TIL counts.	^34^
STING activation in HPV + TSCC, Immunosuppressive cytokine secretion by STING activation, Role of tregs in tumour progression, c-Jun/AP-1 Pathway in CCL22 Induction, Regulation of STING by miR-27	STING (Stimulator of Interferon Genes), Immunosuppressive Cytokines (CCL22, IL-10, IDO), Foxp3, c-Jun (part of AP-1 complex), miR-27a/b	H&E staining, ISH for STING, and p16 IHC to verify the expression and active state of STING are pathological confirmation methods for TSCC samples. The oral squamous cell carcinoma cell lines HSC-3, Scc-4, Scc-1, HB, Scc-14a, Scc-14b, OSCC-3, and Cal-27, as well as the normal human keratinocyte line HaCaT, were cultured in Dulbecco's Modified Eagle's medium with 10 % FBS (Life Technologies, USA) at 37 °C with 5 % CO2 atmosphere. The analysis of gene expression data was normalized through RT-PCR (qRT-PCR) using GAPDH or U6 as reference genes. The Trizol reagent from Invitrogen was used for RNA extraction. The assay for flow cytometry, cell extracts, immunoblotting and cell extracts were prepared. ELISA was used to quantify the amount of secreted CCL22 in cell culture supernatants, Luciferase assay, The Cell Counting Kit-8 (CCK-8) assay, peripheral blood mononuclear cell separation, and the migration assay to gauge the migration of PBMC-derived Tregs towards CCL22-containing supernatants assay were used to assess cell viability. The statistical analysis was done using SPSS 16.0 and GraphPad Prism 5.0.	The levels of STING expression are similar in HPV+ and HPV- TSCC samples. HPV + samples had a higher rate of activated STING. STING was not affecting cell viability or apoptosis when activated in TSCC cell lines (HaCaT, HSC-3, Scc-4). In TSCC cells, STING was activated to secrete cytokines such as CCL22, IDO, and IL-10, all of which are known to promote an immunosuppressive tumour microenvironment. CCL22 is a chemokine that is selectively upregulated by STING. Increased Foxp3+ Tregs infiltration was seen in HPV + TSCC samples. The mediatory of CCL22 by STING activation is done through MAPK/AP-1 pathway, namely c-Jun. Downregulation of c-Jun reduced the expression of CCL22 and Tregs recruitment. In TSCC tissues and cell lines, especially in HPV + samples, miR-27a/b, a microRNA targeting STING is downregulated. Decreased miR-27 levels enhanced STING activation, CCL22 production, Tregs attraction, and immunosuppression.	^49^
Expression of p16, The role of the HPV receptor syndecan-1	p16 (CDKN2A), HPV16 DNA,	p16 Immunohistochemistry (IHC), In Situ Hybridization (ISH)of HPV16 DNA, Histopathological Analysis to confirm the tumour characteristics, statistical analysis by using SPSS version 22	No HPV16 DNA was detected in any of the 109 TSCC samples even though they express p16. HPV16 was not a contributing factor to the majority of TSCC cases 67 % of TSCC cases were p16-negative. 19 % showed weak p16 expression, while 14 % had strong p16 expression. p16 expression did not correlate with age, gender, tumour location, or relapse All TSCC samples analyzed were positive for syndecan-1 HPV does not preferentially infect TSCC. Tonsillar SCC showed a similar syndecan-1 expression to TSCC. Lack of p16 expression in TSCC is associated with worse prognosis, particularly in patients younger than 40 years	^39^
Antigen-Presenting Machinery (APM), APM, The role of CD8^+^ TILs (cytotoxic T lymphocytes)	HPV DNA, p16INK4a, HLA Class I (HC10), TAP1, TAP2, LMP2, LMP7, LMP10, CD44, LRIG1,HLA Class II, HLA-A∗02,	Immunohistochemistry (IHC), HPV DNA Detection by PCR, Histopathological Evaluation to determine tumour size, stage, and nodal involvement, using Stata 13 software for statistical analysis	The model showed acceptable discrimination with an AUC of 0.77 It could reliably differentiate between patients with a high and low risk of relapse or death Age and tumour stage were significant predictors in the model Downregulation or loss of HLA class I expression Low nuclear expression of LMP7	^44^
The function of AP-1, a crucial modulator of HPV carcinogenesis and oncogene expression.	Fos, c-Jun, MMP-9, HPVE6/E7, Fra-1 and p53	HPV genotyping: Preparation of protein extract and electrophoretic mobility shift assay (EMSA): Western blotting, RT-PCR, siRNA transfection and interference assay, In vitro Matrigel cell invasion assay and In vitro cell migration assay.	Shutting down Fra-2 in aggressive tongue tumorigenesis suppressed c-Fos, c-Jun, MMP-9 and HPV E6/E7 proteins, but enhanced Fra-1 and p53. Fra-1 is a transcription factor linked to tumour suppression in this context. P53 is a tumour suppressor protein, often associated with the prevention of aggressive tumorigenesis. The c-Fos/Fra-2 interaction increased with lesion severity. In HPV-negative cases, c-Jun drives poor differentiation and aggressive tumour growth, whereas HPV-positive cases, especially in non-smokers, show well-differentiated tumours with better prognosis. c-Fos, c-Jun, and MMP-9 are key the factors involved in tumour progression and extracellular matrix remodelling. Oncogenic protein expression such as HPV E6/E7 associated with HPV-driven cancers.	^50^
The analysis of 50 paired oral tongue primary tumours included the examination of SNVs, indels, LOH regions and CNVs. The study attempted to link the most important somatic mutations with clinical parameters and epidemiological factors including HPV infection and tumour recurrence.	TP53, CASP8, RASA1, NOTCH and CDKN2A genes, significant amplifications and/or deletions were detected in chromosomes 6–9, and 1 and matrix metalloproteases.	Whole-genome gene expression, SNP genotyping, discovering Copy number Variations (CNVs) and Loss of Heterozygosity (LOH)	A ensemble machine learning method was used to develop a 38 gene minimal signature that is predictive of tumour recurrence. It establishes a link between molecular signatures and clinical and epidemiological factors in a relatively homogeneous tumour population with high HPV prevalence. A minimal set of 38 genes was identified as predictive of tumour recurrence. These genes likely represent alterations in expression or regulation associated with disease progression. The molecular signature is linked to the likelihood of recurrence, suggesting these genes play a role in the aggressive behaviour or survival mechanisms of the tumour.	^55^
53 Greek patients with oral tongue SCC had high-risk HPV E6/E7 mRNA expression and HPV infection.	E6/E7 mRNA	HPV detection, genotyping including HPV genotyping assay and HPV E6/E7 mRNA expression	The E6/E7 mRNA expression rate was 9.4 % while high-risk HPV DNA occurred in 7.5 % of samples and HPV DNA was detected in 11.3 % of oral tongue squamous cell carcinoma cases. The most prevalent genotype was HPV 16. Additionally, 28.3 % of the patients did not smoke or drink. The detection of high-risk HPV E6/E7 mRNA expression makes it plausible that HPV contributes to oral tongue SCC development.	^51^
The study investigated the role of p53, human HPV status, microsatellite instability, epidermal growth factor receptor, and loss of heterozygosity at multiple tumor suppressor loci in oral tongue squamous cell carcinoma.	P53, epidermal growth factor receptor, microsatellite instability	PCR amplification, Microsatellite instability (MSI) screening and loss of heterozygosity (LOH) analysis	The p53 nuclear stability was significantly higher in early-onset squamous cell carcinoma of the oral tongue (SCCOT) than in late-onset. There was a significant correlation between FHIT loss and p53 nuclear stability, especially in those with no history of tobacco use. The poor survival was found to be associated with either p53 nuclear stabilization or p53 mutations in the DNA binding domain. The study also revealed the SCCOT tumorigenesis by age and tobacco use and the potential prognostic value of p53.	^54^
Sort patients in our series of early-stage tongue cancers according to their HPV 16 and p16 status to assess the likelihood of a bad outcome.	HPV 16 and p16	PCR, qPCR and Immunohistochemistry	In this study, the HPV prevalence of 51.2 % was noted in tongue cancers and HPV 16 was detected in 85.2 % of the HPV positive cases. The comparison between HPV 16 DNA and p16 IHC was not very good (kappa <0.2) which raises questions about the validity of using p16 as a surrogate for HPV. The molecular classification also showed that tumours with p16 overexpression were related to a significantly higher risk of death (HR = 2.395; p = 0.005) and disease recurrence (HR = 2.581; p = 0.002), independent of the HPV 16 DNA status. A distinct mechanism for p16 overexpression, independent of HPV infection, is suggested, differing from patterns observed in oropharyngeal cancers.	^14^
Antigen processing machinery (APM) APM	LMP10	HPV Analysis - Bead-based multiplex assay, Cytoplasmic and nuclear LMP10 Analysis- Immunohistochemical staining	In HPV positive cases absence of HLA-A∗02 was associated with better disease-free survival (DFS) but in HPV negative cases it was not.	^43^
Information has not been given	HLA-A∗02	HPV Analysis - Bead-based multiplex assay, HLA-A∗02 Analysis- PCR	In HPV positive cases absence of HLA-A∗02 was associated with better disease-free survival (DFS) but in HPV negative cases it was not.	^45^
Collagen family, cellular growth factor pathways, ECM receptor interaction, ECM structure, and metastasis response.	S100A7, IL1RN, CXCL8, ID1, ECM1, IL6, CLDN4, RAC1, EVPL, TACSTD2, SDC4, DPYSL3, NOS3, EPHB1, LAMC2, CEACAM5, TNC, OLFML2B, PPP1R16B, PFKFB1, ANGPT1, SACS, SCG2, PLCG2, F11R, AGRN, LAMB3, ITGA6, HGF, UBA52, CXCL13, ISLR, COL1A2, MRC1, TPM2, COL7A1, FREM1, VIM, CDK14, PTPRB, COL5A2, SULF1, COL6A1, ACTG2, TIMP2, IL11, CYP1B1, SERPING1, CALCRL, LOXL2, CCBE1, COL6A2, CADM1, SPP1, LUM, AEBP1, COL6A3, POSTN, SPARC, MS4A4A, FN1, COL1A2, COL1A1, COL3A1	Multiplex gene expression assay Immunohistochemical staining	In HPV-positive tumours, 40 genes showed significantly altered expression between High-Grade Dysplasia and invasive cancer. In HPV-negative tumours, 33 genes showed similar differences. HPV+ and HPV-invasive carcinomas show increased activity in collagen family signalling pathways together with ECM receptor interaction, ECM structure, metastasis response and cellular growth factor pathways.	^41^
Antigen Processing Machinery (APM) APM	TAP2, LMP2, and LMP7	Immunohistochemical staining	The TAP2 and LMP7 proteins showed either complete absence or reduced expression in TSCC and BOTSCC samples across all HPV status groups. TAP1 expression correlated with TAP2 expression and LMP2 expression correlated with LMP7 expression. The HLA class I expression showed strong correlation with LMP2 and LMP7 expression which suggests these elements function in antigen presentation.	^46^
The study analyzed TLR4, TLR9, NF-kB/p65, and IFN-β immunohistochemical expression in tumour cells and inflammatory cells as well as normal mucosa cells.	TLR4, TLR9	Immunohistochemical staining and PCR	The expression of TLR4 and TLR9 was higher in tumor and inflammatory cells than in the surrounding mucosa, TLR9 was higher in well-differentiated tumours and TLR4 was negatively correlated with tumor grade. TLR4 and TLR9 were positively correlated with NF-kB and IFN-β levels in malignant tissues, especially in invasive cases. TLR9 and NF-kB levels were higher in HPV positive cases (15.9 % of cases).	^40^

### 3.8 Methods of HPV detection and activity assessment

Among the included studies, only a subset explicitly assessed HPV transcriptional activity or integration using E6/E7 mRNA expression or integration-specific PCR, highlighting that HPV positivity alone does not confirm an active oncogenic role.^34^^,^^52^^,^^51^ Additionally, several studies relied solely on p16 overexpression as a surrogate marker, which may not accurately reflect HPV-driven carcinogenesis in tongue squamous cell carcinoma.^14^^,^^39^^,^^42^^,^^48^

## 4 Discussion

Lip, oral cavity, and oropharyngeal cancers, collectively referred to as oral cancers, represent a significant global health concern, ranking as the 16th most common cancer worldwide, with approximately 389,485 new cases annually.^6^ Within this category, tongue cancer, particularly that affecting the base of the tongue, has gained increasing attention owing to its rising global incidence.^8^ Although alcohol and tobacco use remain the primary risk factors for tongue cancer, the carcinogenic potential of HPV, especially high-risk strains such as HPV-16 and HPV-18, has emerged as a crucial factor in tongue carcinogenesis.^56^ Understanding the molecular mechanisms underlying HPV-induced tongue carcinogenesis has profound implications in clinical practice.^57^ The HPV status of tongue cancer patients serves as a predictive biomarker, informing treatment decisions, and potentially guiding personalized therapeutic approaches.^58^^,^^59^ Advances in molecular biology have paved the way for HPV-targeted treatments, including therapeutic vaccinations and inhibitors of viral oncoproteins.^60^^,^^61^ Moreover, the unique immunogenic profile of HPV-positive tongue cancers has sparked interest in exploring immune checkpoint inhibitors as promising treatment modalities.^62^

In recent years, there has been a surge in studies investigating the molecular mechanisms of HPV-induced tongue carcinogenesis, with a notable peak observed in publications in 2015. Among the studies included in this systematic review, twenty-three were cohort studies, which play a pivotal role in elucidating the complex molecular processes underlying HPV-induced tongue carcinogenesis.^63^^,^^64^ These longitudinal studies provide a systematic framework for monitoring disease progression, allowing researchers to track a defined group of individuals over time and to analyze the genetic, epigenetic, and biochemical changes associated with HPV exposure and subsequent carcinogenesis. These cohort studies offer several advantages. They enable real-time capture of data on viral integration, immune response modulation, and specific gene mutations or expression patterns associated with HPV. This approach helps establish causative links and identify potential biomarkers for early detection and therapeutic targets. Furthermore, cohort studies have provided valuable insights into patient demographics, risk factors, and lifestyle variables, contributing to a comprehensive understanding of tongue cancer development and progression.^65^^,^^66^ By integrating the findings from these cohort studies with those of other research methodologies, scientists can develop a more nuanced understanding of HPV-induced tongue carcinogenesis. This knowledge is crucial for improving the diagnostic accuracy, developing targeted therapies, and ultimately enhancing patient outcomes in the management of HPV-associated tongue cancer.

The review identifies two main pathways through which HPV leads to tongue carcinogenesis which include genetic and epigenetic changes. The process of turning normal cells into cancer cells depends on understanding genetic changes that occur during HPV-induced tongue carcinogenesis.^53^ The HPV virus exists in more than 140 different types which group into two categories of non-cancerous low-risk and cancer-causing high-risk types.^67^ The HPV type 16 represents the most prevalent viral strain which leads to 60–80 % of worldwide head and neck squamous cell carcinoma (HNSCC) cases.^68^ Research shows that HPV type 18 occurs in only 2.8 % of OSCC samples that test positive for HPV but this type appears in 34 % of OSCC and 17 % of laryngeal squamous cell carcinoma.^69^^,^^70^ The clinical and biochemical features of HNSCCs linked to HPV differ from those that do not have HPV infection. Patients who have HPV-positive HNSCC experience better outcomes than those with HPV-negative tumours and HPV-positive cases show lower rates of p53 tumor suppressor gene mutations.^70^, ^71^, ^72^

The research demonstrates that various genes and proteins hold promise as diagnostic tools to study HPV-related tongue cancer development at the molecular level. P16 (CDKN2A) stands out as a commonly investigated biomarker for HPV-related malignancies and functions as one of the primary genes for studying HPV-related tongue carcinogenesis.^73^ The gene functions as the main biomarker for oncogenic activity in high-risk HPVs.^73^ The tumour suppressor protein p16 through CDK-4 and -6 inhibition controls the G1 checkpoint as it regulates these key regulators.^74^ The CDKs phosphorylate retinoblastoma protein (pRb) which causes a structural modification that frees E2F to drive cell cycle progression into the S phase.^75^^,^^76^ Cells gain the ability to keep proliferating after either p16 or pRb becomes inactivated since the G1 checkpoint no longer functions.^77^ The accumulation of P16 occurs in HPV positive tumour cells because cells use it as a compensatory response to Rb inactivation.^78^ Studies have demonstrated that pRb inactivation leads to elevated p16 expression in different cancer types through a reciprocal regulatory mechanism.^79^, ^80^, ^81^, ^82^, ^83^, ^84^, ^85^, ^86^, ^87^, ^88^ Patients who have HPV-related tongue tumours show improved treatment outcomes and better survival rates when their P16 expression levels are elevated compared to HPV-negative patients. P16 functions as both a diagnostic marker for HPV-associated carcinogenesis and as a predictive indicator for personalized therapeutic approaches.^89^^,^^90^

HPV-negative tongue cancers show TP53 mutations frequently but HPV-positive cases exhibit lower mutation rates which indicates different cancer development pathways.^91^ The p53 function gets disrupted either by genetic mutations or by alternative processes including HPV infection.^92^ The E6 protein in HPV-positive cases leads to p53 protein degradation through proteasome pathways.^93^ Considering that the most commonly mutated gene is TP53 in HNSCCs, researchers are investigating how these mutations influence HNSCC progression and treatment response, as well as their potential as prognostic and predictive biomarkers.^94^

In addition, several oncogenes together with tumour suppressor genes have been identified as potential biomarkers. CDC27 together with BCLAF1 and AQP7 show substantial changes in HPV-related tongue cancers, especially CDC27 deletions that correlate with tumour recurrence.^95^ CDC27 functions as a vital part of the anaphase-promoting complex/cyclosome complex and its modified expression patterns can affect cell cycle progression and mitosis and cancer development and patient outcomes.^96^^,^^97^ CDC27 lacks specific targeted treatments in the present clinical setting.^98^ Research indicates that curcumin and miR-27a functionally regulate CDC27.^99^^,^^100^ The nuclear localization of CDC27 prevents its therapeutic use but researchers have identified antibodies against this protein which shows promise for diagnostic purposes.^30^ The cancer research field has extensively studied BCLAF1 as a transcription factor that binds to Bcl2 among the genes that frequently undergo mutations.^101^ BCLAF1 has been identified as a promising candidate for diagnostic and therapeutic development because experimental studies show it contributes to cisplatin resistance in non-small cell lung cancer (NSCLC).^102^^,^^103^

The AQP7 gene encodes a membrane channel which performs known metabolic functions. Research has shown that AQP7 shows overexpression in thyroid cancer while serving as a potential therapeutic target in breast cancer but no studies exist about its function in HNSCC.^104^, ^105^, ^106^ Additional research into AQP7 function is necessary to establish its potential as a biomarker or therapeutic target because tongue cancer shows distinct patterns between HPV-positive and HPV-negative cases.^107^ The gene FGFR3 shows potential as a therapeutic target because it undergoes more significant alterations in HPV-positive HNSCC than in HPV-negative HNSCC. The FGFR3 p.S249C mutation leads to poor patient results mainly in cases of cervical cancer and OPSCC with HPV.^36^^,^^108^, ^109^, ^110^, ^111^ Preclinical and clinical trials of FGFR inhibitors as targeted therapy have produced promising therapeutic results.^112^, ^113^, ^114^, ^115^

Both the viral and host cell genomes undergo a number of epigenetic changes during HPV infection, including ncRNAs production changes, histone modifications, host tumor suppressor genes hypermethylation and hypomethylation or hypermethylation of viral DNA.^116^ The E6 and E7 oncoproteins transactivate the infected cell by engaging and/or altering the activity of a variety of cellular factors that control epigenetic programming.^117^ The alterations made to the chromatin structure as well as the rise in the activity of histone modifying enzymes result in the altered gene expression.^118^^,^^119^ The major causes of the loss of regulation of E6 and E7 expression during HPV infection include the disruption of E1 and/or E2 viral genes during viral DNA integration into the host genome or hypermethylation of the virus's early promoter region within the long control region (LCR), which controls E6 and E7 expression.^120^ Epigenetic pathways are also significant in HPV induced carcinogenesis. Genes like DAPK1, LRPPRC, RAB6C, and ZNF471 are found to be the targets of promoter hypermethylation that leads to the altered gene expression patterns that help in growth of tumours.^116^ The role of epigenetic control in HPV positive tongue cancers is also supported by the existence of the alterations in DNA methylation.^116^, ^117^, ^118^

The non-coding RNAs known as microRNAs (miRNAs) with 18–22 nucleotides in length regulate multiple cellular functions including differentiation, proliferation and survival.^118^ Research has demonstrated that HNSCC patients exhibit altered miRNA expression patterns^118^, ^119^, ^120^, ^121^, ^122^, ^123^ and researchers believe miRNAs contribute to cancer progression and patient survival outcomes.^124^, ^125^, ^126^, ^127^ Additionally, miRNAs have been implicated in the regulation of apoptotic pathways and tumour progression.^128^, ^129^, ^130^ The research by Barker et al.^131^ revealed that HNSCC shows different miRNA profiles across various tissues and sites because anatomical location and tissue type along with tumor etiology significantly influence miRNA expression and distribution. The evaluation of distinct tumor sites requires independent assessment while distinguishing between tumours of different etiologies such as HPV-positive versus HPV-negative tonsillar and base of tongue squamous cell carcinoma (TSCC/BOTSCC) for identifying miRNA signatures as biomarkers in HNSCC.^132^ The insufficient sample size and poor separation of HPV-positive from HPV-negative cases have been identified as factors responsible for the inconsistent miRNA expression patterns reported in HNSCC research.^133^, ^134^, ^135^, ^136^, ^137^, ^138^, ^139^, ^140^ Importantly, miRNAs have emerged as promising prognostic biomarkers according to the information obtained from this systematic review. MiR-185 is linked to worse survival and HPV negative tumours while miR-155 and miR-193b are linked to better survival in HPV positive tumours. These results show that miRNA profiling could be a useful method for prognostic patient stratification in HNSCC.

HPV positive and HPV negative HNSCC vary in the expression of inflammatory markers and immune related proteins in addition to miRNA dysregulation.^141^ Psoriasin (S100A7) is an immunomodulatory protein that is a member of the S100 family and is required for cell survival and maturation due to its calcium binding-motif.^142^ Its overexpression has been linked to clinical outcomes and has been found in premalignant and malignant lesions.^140^, ^141^, ^142^, ^143^, ^144^, ^145^ The expression of Psoriasin was correlated with a worse prognosis in patients with HNSCC in the study by Tripathi et al.^146^ However, this analysis was not done with regard to tumour sub-sites or HPV status. Moreover, immune-related cytokines, such as VEGF, and alterations in antigen-presenting machinery (APM) components have been associated with tumour progression and immune evasion.^146^ These results demonstrate a complex interaction between immune regulation and tumour growth in HNSCC and highlight the importance of differentiating between HPV positive and negative cases when evaluating prognostic factors. While several biomarkers—including p16, FGFR3 mutations, and microRNA profiles—have emerged as promising candidates for stratifying HPV-positive tongue cancers, it is important to acknowledge that these findings are primarily derived from retrospective studies with limited sample sizes and heterogeneous methodologies. As such, their utility in guiding personalized therapy remains speculative. The current evidence base does not support direct clinical implementation, and prospective validation in well-designed longitudinal studies is essential before these biomarkers can be integrated into routine therapeutic decision-making. Therefore, the potential for personalized management should be viewed as a future direction rather than a current clinical reality.

## 5 Conclusion and perspective

HPV-induced tongue carcinogenesis is a multifaceted process involving both genetic and epigenetic alterations that contribute to malignant transformation.^147^ High-risk HPV strains—particularly HPV-16 and HPV-18—promote oncogenesis by inactivating critical tumour suppressor pathways, including p53 and pRb, and by upregulating p16 expression.^148^, ^149^, ^150^ HPV-positive tongue tumours are characterized by distinct epigenetic modifications, such as aberrant DNA methylation patterns, histone alterations, and dysregulation of non-coding RNAs, which collectively modulate immune responses and gene expression.^151^, ^152^ Importantly, these tumours often exhibit different biological behaviour compared to their HPV-negative counterparts, typically being associated with more favourable prognosis and improved treatment responsiveness. Emerging targeted therapies—including HPV-specific vaccines, FGFR inhibitors, and immune checkpoint inhibitors—are being explored as promising strategies for personalized management.^153^, ^154^, ^155^ These approaches may enhance early detection, risk stratification, and therapeutic outcomes based on the disease stage. While current evidence highlights promising biomarkers and therapeutic avenues, further research is warranted to validate these findings and identify additional targets.

Despite these advances, the evidence base remains limited by the retrospective nature of many studies, small and heterogeneous sample sizes, inconsistent follow-up periods, and methodological variability. A notable proportion of included studies originated from a single Swedish centre (Karolinska University Hospital), raising the possibility of geographical and population-specific bias, as well as potential cohort overlap across publications. While we considered performing subgroup or sensitivity analyses to address this, the substantial heterogeneity in study design, outcome reporting, and the absence of comparable quantitative measures precluded formal statistical testing. As this review employed a narrative synthesis rather than a meta-analysis, excluding these studies would not yield a meaningful recalculated effect size. Nevertheless, we acknowledge that certain findings—particularly some biomarker associations, such as FGFR3 mutation patterns and specific microRNA profiles—were predominantly reported in the Swedish cohort and should therefore be interpreted with caution until confirmed in independent populations. Additionally, inconsistent reporting of funding sources across studies limits assessment of potential sponsorship bias. Future research should prioritise rigorous, standardised study designs across diverse populations to validate these observations and inform the development of effective, evidence-based interventions for HPV-associated tongue carcinogenesis.

## Patient's/guardian's consent

This systematic review is based entirely on data from previously published studies and does not involve the collection or reporting of new individual patient data, images, or clinical materials. As such, patient or guardian consent was not required for the conduct of this review.

## Ethics approval and consent to participate

Not Required.

## Consent for publication

All the authors have approved the manuscript for publication.

## Availability of data and materials

The data will be available on reasonable request from the corresponding author.

## Author's contribution

CHK and KS: Conceptualization and designed the study; CHK, MB, KS, SI, KR and YAJ: Analyzed and interpreted the data; CHK, MB, and KS: Wrote the original draft; KKK and RDJ: Project Administration, Resources, Software, Supervision, Writing- Reviewed, and Edited the final draft. All the authors have read and agreed to the published version of the manuscript. Bogahawatte Samarakoon Mudiyanselage Samadarani Siriwardena: Screening and reviewing, Conflict resolution and Supervision, Editing the manuscript

## Ethical clearance

As this study is a systematic review utilizing publicly available data from previously published research, ethical clearance was not required. The review was conducted in accordance with the PRISMA (Preferred Reporting Items for Systematic Reviews and Meta-Analyses) guidelines and adhered to the ethical standards for literature-based research.

## Sources of funding

This systematic review titled *"Molecular Mechanisms of Human Papillomavirus-induced Tongue Carcinogenesis: A Systematic Review"* was conducted without any external funding support. No financial assistance was received from governmental, commercial, or not-for-profit organizations.

## Declaration of competing interest

The authors declare that they have no known competing financial interests or personal relationships that could have appeared to influence the work reported in this paper.

## Abbreviations

MTC -: Mobile Tongue Cancer
OSCC -: Oral Squamous Cell Carcinoma
SCCT -: Squamous Cell Carcinoma of the Tongue
PBMCs -: Peripheral Blood Mononuclear Cells
SCCOC -: Squamous Cell Carcinoma of the Oral Cavity
HNCUP -: Head and Neck Cancer of Unknown Primary
CIS -: Carcinoma in SituCDC27 - Cell Division Cycle 27
BCLAF1 -: Bcl-2-Associated Transcription Factor 1
AQP7 -: Aquaporin 7
BOTSCC -: Base of Tongue Squamous Cell Carcinoma
TSCC -: Tongue Squamous Cell Carcinoma
OPSCC -: Oropharyngeal Squamous Cell Carcinoma
DFS -: Disease-Free Survival, OS - Overall Survival
TNM -: Tumour, Node, Metastasis (cancer staging system)
VEGF -: Vascular Endothelial Growth Factor
IHC -: Immunohistochemistry, PCR - Polymerase Chain Reaction
qPCR -: Quantitative PCR
FFPE -: Formalin-Fixed Paraffin-Embedded
NF-kB -: Nuclear Factor Kappa B
IFN-β -: Interferon Beta
APM -: Antigen Processing Machinery
CNV -: Copy Number Variation
LOH -: Loss of Heterozygosity
CAST-PCR -: Competitive Allele-Specific TaqMan PCR
SDS-PAGE -: Sodium Dodecyl Sulfate–Polyacrylamide Gel Electrophoresis
MALDI-TOF MS -: Matrix-Assisted Laser Desorption/Ionization-Time of Flight Mass Spectrometry
RT-PCR -: Reverse Transcriptase PCR
TAP2 -: Transporter Associated with Antigen Processing 2
LMP2, LMP7 -: Low Molecular Weight Protein 2, 7
TLR4, TLR9 -: Toll-Like Receptor 4, 9
CCL22, IL-10, IDO -: Chemokine (C-C motif) Ligand 22, Interleukin-10, Indoleamine 2,3-Dioxygenase
STING -: Stimulator of Interferon Genes
AP-1 -: Activator Protein 1
miRNA –: MicroRNA
CDKN2A -: Cyclin-Dependent Kinase Inhibitor 2A
