# Supplementary material for: Molecular mechanisms of human papillomavirus-induced tongue carcinogenesis: A systematic review
Source: J Oral Biol Craniofac Res. 2025 Dec 10;16(1):198–221. doi: 10.1016/j.jobcr.2025.11.011 (PMC12755974; doi:10.1016/j.jobcr.2025.11.011)
Supplement: Multimedia component 1 [file mmc1.docx]

**Table S1. The search strings used on “PubMed” database.**

| **Tag** | **Search objectives** | **Search strings** |
| --- | --- | --- |
| #1 | To search for articles on human papillomavirus | (Human papilloma*[Title/Abstract]) OR  (HPV[Title/Abstract]) |
| #2 | To search for articles on the tongue | (Tongue[Title/Abstract]) OR  (oral[Title/Abstract]) |
| #3 | To search for articles on cancer | ((cancer[Title/Abstract]) OR  (carcino*[Title/Abstract])) OR  (malignan*[Title/Abstract]) |
| #4 | To search for articles on molecular mechanisms | ((((((((molecular[Title]) OR  (pathogen*[Title])) OR (oncogen*[Title]))  OR (signal*[Title])) OR (gene*[Title])) OR  (express*[Title])) OR (p53[Title])) OR  (pRb[Title])) OR (pathway[Title]) |
| #5 | To search for articles exploring the molecular mechanisms of human papillomavirus in the pathogenesis of tongue cancer | (((#1) AND (#2)) AND (#3)) AND (#4) |
| ***Filters applied to #5***:  Publication year: from 2014 to 2024  Language of publication: English | |  |

**Table S2. The search strings used on “SCOPUS” database.**

| **Tag** | **Search objectives** | **Search strings** |
| --- | --- | --- |
| #1 | To search for articles on human papillomavirus | ( TITLE-ABS-KEY ( "human papilloma*" ) OR TITLE-ABSKEY ( hpv ) ) |
| #2 | To search for articles on the tongue | ( TITLE-ABS-KEY ( tongue ) OR TITLE-  ABS-KEY ( oral ) ) |
| #3 | To search for articles on cancer | ( TITLE-ABS-KEY ( cancer ) OR TITLE-  ABS-KEY ( carcino* ) OR TITLE-ABSKEY ( malignan* ) ) |
| #4 | To search for articles on molecular mechanisms | ( TITLE-ABS-  KEY ( molecular ) OR TITLE-ABSKEY ( pathogen* ) OR TITLE-ABS-  KEY ( oncogen* ) OR TITLE-ABS-  KEY ( signal* ) OR TITLE-ABS-  KEY ( gene* ) OR TITLE-ABSKEY ( express* ) OR TITLE-ABSKEY ( p53 ) OR TITLE-ABS-  KEY ( prb ) OR TITLE-ABSKEY ( pathway* ) ) |
| #5 | To search for articles exploring the molecular mechanisms of human papillomavirus in the pathogenesis of tongue cancer | ( ( TITLE-ABS-KEY ( "human papilloma*" ) OR TITLE-ABS-KEY ( hpv ) ) ) AND ( ( TITLE-ABS-KEY ( tongue )  OR TITLE-ABS-KEY ( oral ) ) ) AND ( ( |
|  |  | TITLE-ABS-KEY ( cancer ) OR TITLE-  ABS-KEY ( carcino* ) OR TITLE-ABS-  KEY ( malignan* ) ) ) AND ( ( TITLEABS-KEY ( molecular ) OR TITLE-ABSKEY ( pathogen* ) OR TITLE-ABS-KEY ( oncogen* ) OR TITLE-ABS-KEY ( signal* ) OR TITLE-ABS-KEY ( gene* ) OR  TITLE-ABS-KEY ( express* ) OR TITLEABS-KEY ( p53 ) OR TITLE-ABS-KEY ( prb ) OR TITLE-ABS-KEY ( pathway* ) )  ) |
| ***Filters applied to #5***:  Publication year: from 2014 to 2024  Language of publication: English  Document type: Article | | |

**Table S3. The search strings used on “Web of Science” database.**

| **Tag** | **Search objectives** | **Search strings** |
| --- | --- | --- |
| #1 | To search for articles on human papillomavirus | (AB=(Human papilloma*)) OR AB=(HPV) |
| #2 | To search for articles on the tongue | (AB=(Tongue)) OR AB=(oral) |
| #3 | To search for articles on cancer | ((AB=(cancer)) OR AB=(carcino*)) OR  AB=(malignan*) |
| #4 | To search for articles on molecular mechanisms | ((((((((AB=(molecular)) OR  AB=(pathogen*)) OR AB=(oncogen*)) OR  AB=(signal*)) OR AB=(gene*)) OR  AB=(express*)) OR AB=(p53)) OR  AB=(pRb)) OR AB=(pathway) |
| #5 | To search for articles exploring the molecular mechanisms of human papillomavirus in the pathogenesis of tongue cancer | #1 AND #2 AND #3 AND #4 |
| ***Filters applied to #5***:  Publication year: from 2014 to 2024  Language of publication: English  Document type: Article | |  |

**Table S4. The search strings used on “Dentistry & Oral Sciences Source” and “AMED – The Allied and Complementary Medicine Database” databases via EBSCOHost interface.**

| **Tag** | **Search objectives** | **Search strings** |
| --- | --- | --- |
| S1 | To search for articles on human papillomavirus | AB Human Papilloma* OR AB HPV |
| S2 | To search for articles on the tongue | AB Tongue OR AB Oral |
| S3 | To search for articles on cancer | AB Cancer OR AB Carcino* OR AB Malignan* |
| S4 | To search for articles on molecular mechanisms | AB Molecular OR AB Pathogen* OR AB  Oncogen* OR AB Signal* OR AB Gene*  OR AB Express* OR AB P53 OR AB pRb  OR AB Pathway |
| S5 | To search for articles exploring the molecular mechanisms of human papillomavirus in the pathogenesis of tongue cancer | S1 AND S2 AND S3 AND S4 |
| ***Filters applied to #5***:  Publication year: from 2014 to 2024  Language of publication: English  Source type: Academic journals | |  |

**Table S5. List of literature considered for full text screening and their screening outcomes**

| **No.** | **Bibliometric information** |  | **Included** | **Excluded (with Reasons)** |
| --- | --- | --- | --- | --- |
| **Literature obtained from database search** | | |  | |
| 1 | Maléřová S, Kalfeřt D, Grega M, Tachezy R, Klozar J. The significance of p16 protein expression in oral squamous cell carcinoma. Epidemiologie,  Mikrobiologie, Imunologie: Casopis Spolecnosti pro Epidemiologii a Mikrobiologii Ceske Lekarske Spolecnosti JE Purkyne. 2020 Jan 1;69(2):64-72. |  |  | Yes (Foreign language) |
| 2 | Adduri, R. S., Kotapalli, V., Gupta, N. A., Gowrishankar, S., Srinivasulu,  M., Ali, M. M., Rao, S., Uppin, S. G., Nayak, U. K., Dhagam, S.,  Chigurupati, M. V., & Bashyam, M. D. (2014). undefined. *BMC Clinical*  *Pathology*, *14*(1). <https://doi.org/10.1186/1472-6890-14-37> | Yes |  |  |
| 3 | Bersani, C., Mints, M., Tertipis, N., Haeggblom, L., Näsman, A.,  Romanitan, M., Dalianis, T., & Ramqvist, T. (2018). Microrna-155, -185 and -193b as biomarkers in human papillomavirus positive and negative tonsillar and base of tongue squamous cell carcinoma. *Oral Oncology*, *82*, 8-16. <https://doi.org/10.1016/j.oraloncology.2018.04.021> | Yes |  |  |
| 4 | Bersani, C., Sivars, L., Haeggblom, L., DiLorenzo, S., Mints, M., ÄhrlundRichter, A., Tertipis, N., Munck-Wikland, E., Näsman, A., Ramqvist, T., & Dalianis, T. (2017). Targeted sequencing of tonsillar and base of tongue cancer and human papillomavirus positive unknown primary of the head and neck reveals prognostic effects of mutated FGFR3. *Oncotarget*, *8*(21),  35339-35350. <https://doi.org/10.18632/oncotarget.15240> | Yes |  |  |
| 5 | Bhat, S., Kabekkodu, S. P., Jayaprakash, C., Radhakrishnan, R., Ray, S., & Satyamoorthy, K. (2017). Gene promoter-associated CPG island hypermethylation in squamous cell carcinoma of the tongue. *Virchows Archiv*, *470*(4), 445-454. <https://doi.org/10.1007/s00428-017-2094-2> | Yes |  |  |
| 6 | Garnaes, E., Frederiksen, K., Kiss, K., Andersen, L., Therkildsen, M. H.,  Franzmann, M. B., Specht, L., Andersen, E., Norrild, B., Kjaer, S. K., &  Von Buchwald, C. (2016). Double positivity for HPV DNA/p16 in tonsillar | Yes |  |  |

|  | and base of tongue cancer improves prognostication: Insights from a large population‐based study. *International Journal of Cancer*, *139*(11), 25982605. <https://doi.org/10.1002/ijc.30389> |  |  |
| --- | --- | --- | --- |
| 7 | Gupta, S., Kumar, P., Kaur, H., Sharma, N., Saluja, D., Bharti, A. C., & Das, B. C. (2015). Selective participation of c-Jun with Fra-2/C-fos promotes aggressive tumor phenotypes and poor prognosis in tongue cancer.  *Scientific Reports*, *5*(1). <https://doi.org/10.1038/srep16811> | Yes |  |
| 8 | Gupta, S., Kumar, P., Kaur, H., Sharma, N., Gupta, S., Saluja, D., Bharti, A. C., & Das, B. (2018). Constitutive activation and overexpression of NFκb/C-rel in conjunction with p50 contribute to aggressive tongue tumorigenesis. *Oncotarget*, *9*(68), 33011-33029. <https://doi.org/10.18632/oncotarget.26041> | Yes |  |
| 9 | Khowal, S., Naqvi, S. H., Monga, S., Jain, S. K., & Wajid, S. (2018). Assessment of cellular and serum proteome from tongue squamous cell carcinoma patient lacking addictive proclivities for tobacco, betel nut, and alcohol: Case study. *Journal of Cellular Biochemistry*, *119*(7), 5186-5221. <https://doi.org/10.1002/jcb.26554> | Yes |  |
| 10 | Krishnan, N. M., Gupta, S., Palve, V., Varghese, L., Pattnaik, S., Jain, P., Khyriem, C., Hariharan, A., Dhas, K., Nair, J., Pareek, M., Prasad, V., Siddappa, G., Suresh, A., Kekatpure, V., Kuriakose, M., & Panda, B. (2015). Integrated analysis of oral tongue squamous cell carcinoma identifies key variants and pathways linked to risk habits, HPV, clinical parameters and tumor recurrence. *F1000Research*, *4*, 1215. <https://doi.org/10.12688/f1000research.7302.1> | Yes |  |
| 11 | Liang, D., Xiao-Feng, H., Guan-Jun, D., Er-Ling, H., Sheng, C., Ting-Ting, W., Qin-Gang, H., Yan-Hong, N., & Ya-Yi, H. (2015). Activated STING enhances Tregs infiltration in the HPV-related carcinogenesis of tongue squamous cells via the c-Jun/CCL22 signal. *Biochimica et Biophysica Acta (BBA) - Molecular Basis of Disease*, *1852*(11), 2494-2503. <https://doi.org/10.1016/j.bbadis.2015.08.011> | Yes |  |
| 12 | Marklund, L., Holzhauser, S., de Flon, C., Zupancic, M., Landin, D., Kolev, A., Haeggblom, L., Munck-Wikland, E., Hammarstedt-Nordenvall, L., | Yes |  |

|  | Dalianis, T., & Näsman, A. (2020). Survival of patients with oropharyngeal squamous cell carcinomas (OPSCC) in relation to TNM 8 – Risk of incorrect downstaging of HPV-mediated non-tonsillar, non-base of tongue carcinomas. *European Journal of Cancer*, *139*, 192-200. <https://doi.org/10.1016/j.ejca.2020.08.003> |  |  |
| --- | --- | --- | --- |
| 13 | Minami, K., Kogashiwa, Y., Ebihara, Y., Nakahira, M., Sugasawa, M., Fujino, T., & Yasuda, M. (2017). Human papillomavirus and p16 protein expression as prognostic biomarkers in mobile tongue cancer. *Acta OtoLaryngologica*, *137*(10), 1121-1126. <https://doi.org/10.1080/00016489.2017.1339327> | Yes |  |
| 14 | Ramqvist, T., Mints, M., Tertipis, N., Näsman, A., Romanitan, M., &  Dalianis, T. (2015). Studies on human papillomavirus (HPV) 16 E2, E5 and E7 mRNA in HPV-positive tonsillar and base of tongue cancer in relation to clinical outcome and immunological parameters. *Oral Oncology*, *51*(12), 1126-1131. <https://doi.org/10.1016/j.oraloncology.2015.09.007> | Yes |  |
| 15 | Ramqvist, T., Näsman, A., Franzén, B., Bersani, C., Alexeyenko, A.,  Becker, S., Haeggblom, L., Kolev, A., Dalianis, T., & Munck-Wikland, E. (2018). Protein expression in tonsillar and base of tongue cancer and in relation to human papillomavirus (HPV) and clinical outcome. *International Journal of Molecular Sciences*, *19*(4), 978.  <https://doi.org/10.3390/ijms19040978> | Yes |  |
| 16 | Ramshankar, V., Soundara, V. T., Shyamsundar, V., Ramani, P., & Krishnamurthy, A. (2014). Risk stratification of early stage oral tongue cancers based on HPV status and p16 Immunoexpression. *Asian Pacific Journal of Cancer Prevention*, *15*(19), 8351-8359.  <https://doi.org/10.7314/apjcp.2014.15.19.8351> | Yes |  |
| 17 | Sgaramella, N., Coates, P. J., Strindlund, K., Loljung, L., Colella, G.,  Laurell, G., Rossiello, R., Muzio, L. L., Loizou, C., Tartaro, G., Olofsson, K., Danielsson, K., Fåhraeus, R., & Nylander, K. (2015). Expression of p16 in squamous cell carcinoma of the mobile tongue is independent of HPV infection despite presence of the HPV-receptor syndecan-1. *British Journal of Cancer*, *113*(2), 321-326. <https://doi.org/10.1038/bjc.2015.207> | Yes |  |

| 18 | Tertipis, N., Haeggblom, L., Nordfors, C., Grün, N., Näsman, A., Vlastos, A., Dalianis, T., & Ramqvist, T. (2014). Correlation of LMP10 expression and clinical outcome in human papillomavirus (HPV) positive and HPVnegative tonsillar and base of tongue cancer. *PLoS ONE*, *9*(4), e95624.  <https://doi.org/10.1371/journal.pone.0095624> | Yes |  |
| --- | --- | --- | --- |
| 19 | Tertipis, N., Haeggblom, L., Grün, N., Nordfors, C., Näsman, A., Dalianis, T., & Ramqvist, T. (2015). Reduced expression of the antigen processing machinery components TAP2, LMP2, and LMP7 in tonsillar and base of tongue cancer and implications for clinical outcome. *Translational Oncology*, *8*(1), 10-17. <https://doi.org/10.1016/j.tranon.2014.11.002> | Yes |  |
| 20 | Tertipis, N., Hammar, U., Näsman, A., Vlastos, A., Nordfors, C., Grün, N.,  Ährlund-Richter, A., Sivars, L., Haeggblom, L., Marklund, L.,  Hammarstedt-Nordenvall, L., Chaturvedi, A. K., Munck-Wikland, E., Ramqvist, T., Bottai, M., & Dalianis, T. (2015). A model for predicting clinical outcome in patients with human papillomavirus-positive tonsillar and base of tongue cancer. *European Journal of Cancer*, *51*(12), 1580-1587. <https://doi.org/10.1016/j.ejca.2015.04.024> | Yes |  |
| 21 | Zafereo, M. E., Xu, L., Dahlstrom, K. R., Viamonte, C. A., El-Naggar, A. K., Wei, Q., Li, G., & Sturgis, E. M. (2016). Squamous cell carcinoma of the oral cavity often overexpresses p16 but is rarely driven by human papillomavirus. *Oral Oncology*, *56*, 47-53. <https://doi.org/10.1016/j.oraloncology.2016.03.003> | Yes |  |
| 22 | Zupancic, M., Haeggblom, L., Landin, D., Marklund, L., Dalianis, T., & Näsman, A. (2021). Psoriasin expression is associated with survival in patients with human papillomavirus-positive base of tongue squamous cell carcinoma. *Oncology Letters*, *21*(4). <https://doi.org/10.3892/ol.2021.12538> | Yes |  |
| 23 | Ährlund-Richter, A., Holzhauser, S., Dalianis, T., Näsman, A., & Mints, M. (2021). Whole-exome sequencing of HPV positive tonsillar and base of tongue squamous cell carcinomas reveals a global mutational pattern along with relapse-specific somatic variants. Cancers, 14(1), 77. <https://doi.org/10.3390/cancers14010077> | Yes |  |
| 24 | Tsimplaki E, Argyri E, Xesfyngi D, Daskalopoulou D, Stravopodis DJ, Panotopoulou E. Prevalence and expression of human papillomavirus in 53 patients with oral tongue squamous cell carcinoma. Anticancer Res. 2014 Feb;34(2):1021-5. PMID: 24511049. | Yes |  |
| 25 | Dong Y, Pan J, Ni Y, Huang X, Chen X, Wang J. High expression of EphB6  protein in tongue squamous cell carcinoma is associated with a poor outcome. International Journal of Clinical and Experimental Pathology. 2015;8(9):11428. |  | Yes (Does not address or include content relevant to HPV ) |
| 26 | Bersani, C.; Haeggblom, L.; Ursu, R.G.; Giusca, S.E.; Marklund, L.; Ramqvist, T.; Nasman, A.; Dalianis, T. Overexpression of FGFR3 in HPVpositive Tonsillar and Base of Tongue Cancer Is Correlated to Outcome.  Anticancer Res. 2018, 38, 4683–4690. | Yes |  |
| 27 | Daskalopoulos, A. G., Avgoustidis, D., Chaisuparat, R., Karanikou, M.,  Lazaris, A. C., Sklavounou, A., & Nikitakis, N. G. (2020). Assessment of TLR4 and TLR9 signaling and correlation with human papillomavirus status and histopathologic parameters in oral tongue squamous cell carcinoma. *Oral Surgery, Oral Medicine, Oral Pathology and Oral Radiology*, *129*(5), 493-513. | Yes |  |
|  | **Literature obtained from manual searches of the included literature** | |  |
| 1 | Tertipis, N.; Villabona, L.; Nordfors, C.; Nasman, A.; Ramqvist, T.; Vlastos, A.; Masucci, G.; Dalianis, T. HLA-A*02 in relation to outcome in human papillomavirus positive tonsillar and base of tongue cancer.  Anticancer Res. 2014, 34, 2369–2375. | Yes |  |
| 2 | Haeggblom L, Ährlund-Richter A, Mirzaie L, Farrajota Neves da Silva P, Ursu RG, Ramqvist T and Näsman A: Differences in gene expression between high-grade dysplasia and invasive HPV+ and HPV- tonsillar and base of tongue cancer. Cancer Med 8: 6221-6232, 2019 | Yes |  |

**Table S6. Quality appraisal outcomes of the appraised quantitative randomized studies using the Mixed Methods Appraisal Tool**

| **No.** | **Author (Year)** | **Study Design** | **Responses to the Appraisal Questions for Quantitative Randomized Studies** | | | | | |  | **Scored**  **Points (out of a Total of 7 Points)** | **Grade** |
| --- | --- | --- | --- | --- | --- | --- | --- | --- | --- | --- | --- |
|  |  |  | Are there clear research questions? | Do the collected data allow to address the research questions? | Is randomization appropriately performed? | Are the groups comparable at baseline? | Are there complete outcome data? | Are outcome assessors blinded to the intervention provided? | Did the participants adhere to the assigned intervention? |  |  |
|  | Nil | Nil | Nil | Nil | Nil | Nil | Nil | Nil | Nil | Nil | Nil |
| Yes – 1 point; No – 0 point; I can’t tell – 0 point; Above average – 4/7 points and above; Below average – 3/7 points and below | | | | | | | | |  |  |  |

**Table S7. Quality appraisal outcomes of the appraised quantitative non-randomized studies using the Mixed Methods Appraisal Tool**

| **No.** | **Author (Year)** | **Study Design** | **Responses to the Appraisal Questions for Quantitative Non-randomized Studies** | | | | | | | **Scored**  **Points (out of a Total of 7 Points)** | **Grade** |
| --- | --- | --- | --- | --- | --- | --- | --- | --- | --- | --- | --- |
|  |  |  | Are there clear research questions ? | Do the collected data allow to address the research questions? | Are the participants representative of the largest population? | Are measurements appropriate regarding both the outcome and intervention (or exposure)? | Are there complete outcome data? | Are the confounders accounted for in the design and analysis? | During the study period, is the intervention administered (or exposure occurred) as intended? |  |  |
| 1. | ÄhrlundRichter et al. (2021) | Cohort study (Retrospective) | Yes | Yes | Can’t tell | Yes | Yes | Yes | Yes | 6/7 | Above  average |
| 2. | Zupancic et al.,  (2021) | Cohort Study (Retrospective) | Yes | Yes | No | Yes | Yes | Yes | Yes | 6/7 | Above Average |
| 3. | Marklund et al., (2020) | Cohort study (Retrospective) | Yes | Yes | Yes | Yes | Yes | Yes | Yes | 7/7 | Above Average |
| 4. | Bersani et al., (2018a) | Cohort study (Retrospective) | Yes | Yes | No | Yes | Yes | Yes | Yes | 6/7 | Above Average |
| 5. | Ramqvist et al. (2018) | Cohort study  (Retrospective) | Yes | Yes | No | Yes | Yes | Yes | Yes | 6/7 | Above average |
| 6. | Bersani et al. (2018b) | Cohort study  (Retrospective) | Yes | Yes | No | Yes | Yes | Yes | Yes | 6/7 | Above average |
| 7. | Khowal et al. (2018) | Case-control study | Yes | Yes | No | Yes | Yes | Yes | Yes | 6/7 | Above Average |
| 8. | Gupta et al.  (2018) | Case-control study | Yes | Yes | No | Yes | Yes | Yes | Yes | 6/7 | Above Average |
| 9. | Minami et al. (2017) | Cohort study  (Retrospective) | Yes | Yes | No | Yes | Yes | Yes | Yes | 6/7 | Above average |
| 10. | Bhat et al.  (2017) | Cohort study (Prospective) | Yes | Yes | can't tell | Yes | Yes | No | Yes | 5/7 | Above Average |
| 11. | Bersani et al. (2017) | Cohort Study  (Retrospective) | Yes | Yes | Yes | Yes | Can’t tell | Yes | Can’t tell | 5/7 | Above Average |
| 12. | Zafereo et al., (2016) | Cohort study  (Prospective) | Yes | Yes | I can’t tell | Yes | I can’t tell | Yes | I can’t tell | 4/7 | Above Average |
| 13. | Garnaes et al. (2016) | Cohort study  (Retrospective) | Yes | Yes | Yes | Yes | Yes | Yes | I can’t tell | 6/7 | Above Average |
| 14. | Ramqvist et al. (2015) | Cohort Study (Retrospective) | Yes | Yes | I can’t tell | Yes | Yes | I can’t tell | I can’t tell | 4/7 | Above Average |
| 15. | Liang et al.  (2015) | Cohort Study  (Prospective) | Yes | Yes | I can’t tell | I can’t tell | Yes | I can’t tell | I can’t tell | 3/7 | Below Average |
| 16. | Sgaramella et  al., (2015) | Cohort Study  (Retrospective) | Yes | Yes | I can’t tell | Yes | Yes | Yes | I can’t tell | 5/7 | Above Average |
| 17. | Tertipis et al. (2015a) | Cohort Study  (Retrospective) | Yes | Yes | I can’t tell | Yes | Yes | Yes | I can’t tell | 5/7 | Above Average |
| 18. | Gupta et al.  (2015) | Cohort study  (Prospective) | Yes | Yes | Yes | Yes | Yes | No | Yes | 6/7 | Above Average |
| 19. | Panda et al.  (2015) | Case-control study | Yes | Yes | Yes | Yes | I can’t tell | No | Yes | 5/7 | Above Average |
| 20. | Tsimplaki et al. (2014) | Cohort study  (Prospective) | Yes | Yes | I can’t tell | Yes | Yes | Yes | I can’t tell | 5/7 | Above Average |
| 21. | Adduri et al. (2014) | Case-control study | Yes | Yes | I can’t tell | Yes | Yes | I can’t tell | Yes | 5/7 | Above Average |
| 22. | Ramshankar et  al. (2014) | Cohort study  (Retrospective) | Yes | Yes | I can’t tell | Yes | Yes | No | Yes | 5/7 | Above Average |
| 23. | Tertipis et al. (2014a) | Cohort study (Retrospective) | Yes | Yes | Yes | I can’t tell | Yes | Yes | I can’t tell | 5/7 | Above Average |
| 24. | Tertipis et al. (2014b) | Cohort study (Retrospective) | Yes | Yes | I can’t tell | Yes | Yes | Yes | Yes | 6/7 | Above Average |
| 25. | Haeggblom et  al. (2019) | Cohort study (Retrospective) | Yes | Yes | No | Yes | Yes | Yes | Yes | 7/7 | Above Average |
| 26. | Tertipis et al. (2015b) | Cohort study (Retrospective) | Yes | Yes | I can’t tell | I can’t tell | Yes | Yes | Yes | 5/7 | Above Average |
| 27 | Daskalopoulos et al. (2020) | Cohort study (Retrospective) | Yes | Yes | No | Yes | Yes | No | Yes | 5/7 | Above Average |
| Yes – 1 point; No – 0 point; I can’t tell – 0 point; Above average – 4/7 points and above; Below average – 3/7 points and below | | | | | | | | |  |  |  |

**Table S8. Quality appraisal outcomes of the appraised quantitative descriptive study using the Mixed Methods Appraisal Tool**

| **No.** | **Author (Year)** | **Study Design** | **Responses to the Appraisal Questions for Quantitative Descriptive Studies** | | | | | |  | **Scored**  **Points**  **(out of a Total of**  **7 Points)** | **Grade** |
| --- | --- | --- | --- | --- | --- | --- | --- | --- | --- | --- | --- |
|  |  |  | Are there clear research questions? | Do the collected data allow to address the research questions? | Is the sampling strategy relevant to address the research question? | Is the sample representative of the target population? | Are the measurements appropriate? | Is the risk of nonresponse bias low? | Is the statistical analysis appropriate to answer the research question? |  |  |
|  | Nil | Nil | Nil | Nil | Nil | Nil | Nil | Nil | Nil | Nil | Nil |
| Yes – 1 point; No – 0 point; I can’t tell – 0 point; Above average – 4/7 points and above; Below average – 3/7 points and below | | | | | | | | |  |  |  |

**Table S9. Quality appraisal outcomes of the appraised qualitative study using the Mixed Methods Appraisal Tool**

| **No.** | **Author (Year)** | **Study Design** | **Responses to the Appraisal Questions for Qualitative Studies** | | | | | |  | **Scored**  **Points**  **(out of a Total of**  **7 Points)** | **Grade** |
| --- | --- | --- | --- | --- | --- | --- | --- | --- | --- | --- | --- |
|  |  |  | Are there clear research questions? | Do the collected data allow to address the research questions? | Is the qualitative approach appropriate to answer the research question? | Are the qualitative data collection methods adequate to address the research question? | Are the findings adequately derived from the data? | Is the  interpretation of results sufficiently substantiated by data? | Is there coherence between qualitative data sources, collection, analysis and interpretation? |  |  |
|  | Nil | Nil | Nil | Nil | Nil | Nil | Nil | Nil | Nil | Nil | Nil |
| Yes – 1 point; No – 0 point; I can’t tell – 0 point; Above average – 4/7 points and above; Below average – 3/7 points and below | | | | | | | | |  |  | |
